# Exploring the potential of some natural indoles as antiviral agents: quantum chemical analysis, inverse molecular docking, and affinity calculations

**DOI:** 10.3389/fchem.2024.1521298

**Published:** 2025-01-16

**Authors:** Amany Belal, Aly Abdou, Samar F. Miski, Mohamed A. M. Ali, Heba I. Ghamry, Ahmad J. Obaidullah, Mohamed Y. Zaky, Ahmed H. E. Hassan, Eun Joo Roh, Ahmed A. Al-Karmalawy, Mona H. Ibrahim

**Affiliations:** ^1^ Department of Pharmaceutical Chemistry, College of Pharmacy, Taif University, Taif, Saudi Arabia; ^2^ Chemistry Department, Faculty of science, Sohag university, Sohag, Egypt; ^3^ Pharmacology and Toxicology Department, College of Pharmacy, Taibah University, Madinah, Saudi Arabia; ^4^ Department of Biology, College of Science, Imam Mohammad Ibn Saud Islamic University (IMSIU), Riyadh, Saudi Arabia; ^5^ Nutrition and Food Science, Department of Biology, College of Science, King Khalid University, Abha, Saudi Arabia; ^6^ Department of Pharmaceutical Chemistry, College of Pharmacy, King Saud University, Riyadh, Saudi Arabia; ^7^ Drug Exploration and Development Chair (DEDC), Department of Pharmaceutical Chemistry, College of Pharmacy, King Saudi University, Riyadh, Saudi Arabia; ^8^ Molecular Physiology Division, Zoology Department, Faculty of Science, Beni-Suef University, BeniSuef, Egypt; ^9^ Medicinal Chemistry Laboratory, Department of Pharmacy, College of Pharmacy, Kyung Hee University, Seoul, Republic of Korea; ^10^ Department of Medicinal Chemistry, Faculty of Pharmacy, Mansoura University, Mansoura, Egypt; ^11^ Chemical and Biological Integrative Research Center, Korea Institute of Science and Technology (KIST), Seoul, Republic of Korea; ^12^ Division of Bio-Medical Science & Technology, University of Science and Technology, Daejeon, Republic of Korea; ^13^ Department of Pharmaceutical Chemistry, College of Pharmacy, The University of Mashreq, Baghdad, Iraq; ^14^ Department of Pharmaceutical Chemistry, Faculty of Pharmacy, Horus University-Egypt, New Damietta, Egypt; ^15^ Department of Pharmaceutical Medicinal Chemistry and Drug Design, Faculty of Pharmacy (Girls), Al-Azhar University, Cairo, Egypt

**Keywords:** indole alkaloids, HCV, HIV, DFT, NBO analysis

## Abstract

Human immunodeficiency virus (HIV) and hepatitis C virus (HCV) infections represent critical global health challenges due to the high morbidity and mortality associated with co-infections. HIV, the causative agent of acquired immunodeficiency syndrome (AIDS), infects 4,000 people daily, potentially leading to 1.2 million new cases by 2025, while HCV chronically affects 58 million people, causing cirrhosis and hepatocellular carcinoma. Indole-based compounds play a crucial role in antiviral drug development due to their “privileged scaffold” structure. This study investigates the antiviral potential of natural indoles, gardflorine A–C, derived from *Gardneria multiflora* Makino, a plant traditionally used to treat various ailments. We employed molecular docking, ADMET analysis, and computational techniques [frontier molecular orbital (FMO), natural bond orbital (NBO), and density functional theory (DFT)] to evaluate these compounds” potential as multi-target antiviral agents against HIV and HCV proteins.

## 1 Introduction

**FIGURE 1:**
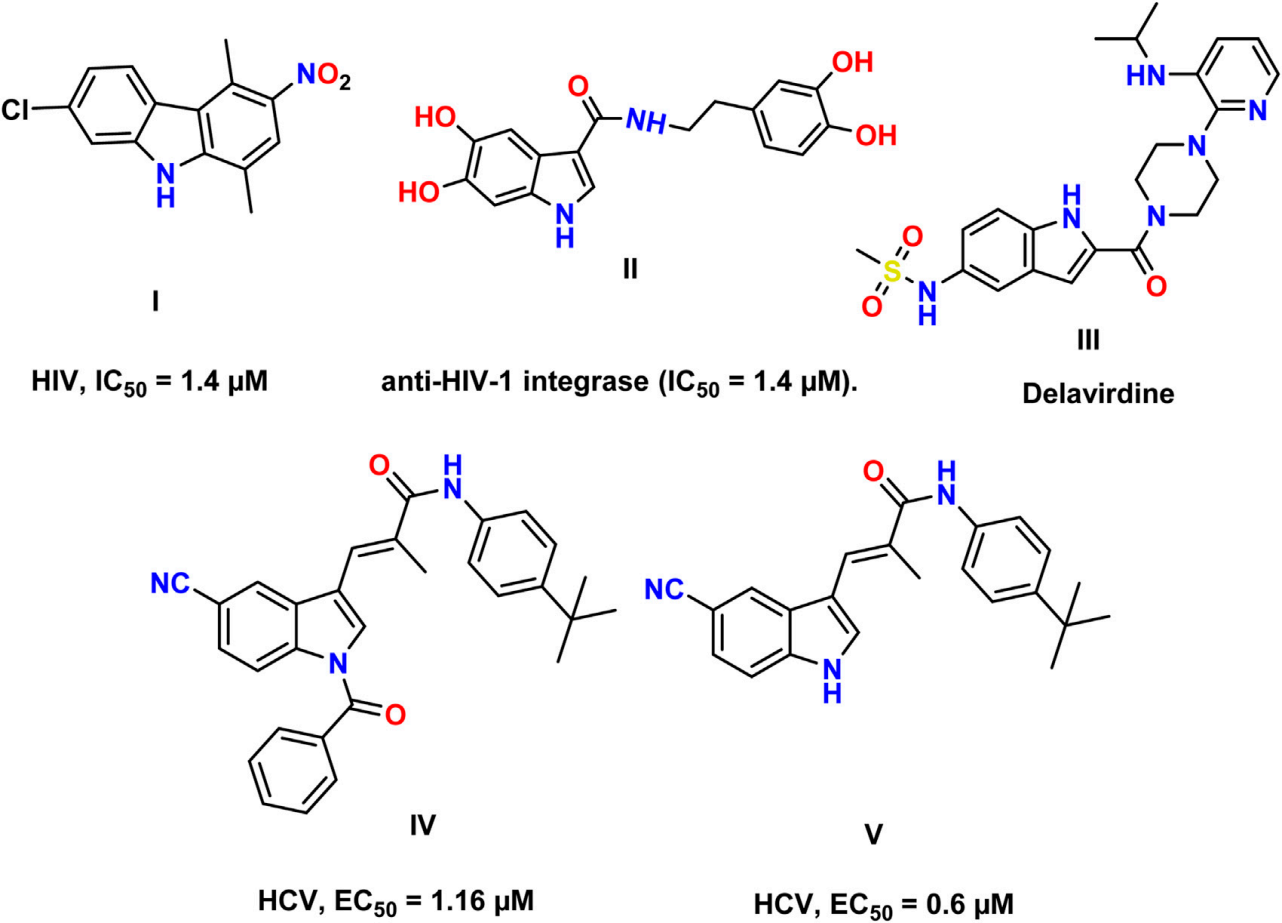
Reported indole compounds I, II, and III as anti-HIV and IV, and V as anti-HCV agents.

Human immunodeficiency virus (HIV) and hepatitis C virus (HCV) infections have emerged as pressing global public health concerns. Even more striking is the quick emergence of HIV–HCV co-infection as a leading cause of illness and mortality (Akhtar et al., 2022). Acquired immunodeficiency syndrome (AIDS) is caused by HIV, which infects 4,000 people daily. If current trends continue, 1.2 million people will be newly infected with HIV in 2025, which is three times the target of 370,000 new infections set for 2025 (2022-global-aids-update-summary_en, 2022). Currently, 58 million people have chronic hepatitis C virus infection, and yearly, there are approximately 1.5 million new cases. Cirrhosis and hepatocellular carcinoma were the primary causes of death associated with hepatitis C in 2019, as estimated by the World Health Organization (Parsons, 2022; Wang et al., 2024; Duan et al., 2024). HIV belongs to the *Lentivirus* genus within the Orthoretrovirinae subfamily of the Retroviridae family. HIV is divided into types 1 and 2 based on genetic traits and antigenic distinctions (HIV-1 and HIV-2) (Seitz, 2016). The HIV genome is composed of two identical single-stranded RNA molecules encased within the virus particle’s nucleus. The HIV provirus genome, also known as proviral DNA, is produced via reverse transcription of the viral RNA genome into DNA, destruction of the RNA, and integration of double-stranded HIV DNA into the human genome (Seitz, 2016; Hu et al., 2022; Kang et al., 2018). The initial stages of cell infection are characterized by intricate protein–protein interactions (PPIs). The mature HIV particle’s surface glycoprotein gp120 interacts with the specific receptors of the host organism (Sundquist and Kräusslich, 2012; Li et al., 2018; Wang et al., 2024). Afterward, the fusion of the cell membrane and viral envelope is accomplished. The fusion of the viral and cellular membranes causes the translocation of the viral capsid into the cytoplasm. The endosome absorbs the capsid, and a change in pH in the phagosome triggers the release of the capsid’s contents into the cytoplasm (Seitz, 2016; Lou et al., 2023). The activation of reverse transcriptase (RT) occurs in the cytoplasm. HIV RT transfers the HIV genome from single-stranded RNA to complementary DNA. Parallel to DNA synthesis, RNase H degrades the RNA strand enzymatically. Then, the DNA-dependent DNA polymerase activity of RT converts single-stranded cDNA into double-stranded DNA (proviral DNA) (Isel et al., 2010). This DNA is transported into the cell nucleus by integrase (IN) and linear or circular proviral DNA via nucleopores. Integrase then randomly integrates the proviral genome into the genome of the human host cell. The incorporation of proviral DNA completes the HIV infection process within the cell, establishing a persistent infection (Di Santo, 2014). Yet, following the activation of infected cells, the LTR promoter of the proviral genome can serve as an attachment point for cellular DNA-dependent RNA polymerases and several transcription factors that initiate the synthesis of viral mRNA and genomic RNA (Rampersad and Tennant, 2018). The main target proteins in HIV/AIDS treatment are reverse transcriptase, protease, and integrase, which suppress viral replication below detectable levels (Arhel and Kirchhoff, 2010). HCV is associated with a high incidence of liver disorders and poses a significant hazard to public health. HCV encodes a single polyprotein; the HCV viral structure consists of envelope glycoproteins in a lipid bilayer containing the viral core protein and RNA (Li and Lo, 2015; Morozov and Lagaye, 2018). Viral RNA is translated by host machinery into a polyprotein, which is cleaved by host and viral-encoded proteases into 10 mature viral proteins, along with several nonstructural (NS) proteins, following cell entrance (Bonamassa et al., 2015). A complex of two viral proteases, NS3 and NS4A proteins, is involved in post-translational processing. NS3 is responsible for proteolytic activity, while NS4A, a membrane protein, acts as a cofactor (Lin). A highly structured replication complex composed of NS3, NS4A, NS4B, NS5A, and NS5B synthesizes new viral RNA. NS5B is an RNA-dependent RNA polymerase required for viral replication. NS5A has a putative involvement in the formation of the replication complex and in controlling replication (Romero-Brey and Lohmann, 2016). It also participates in the assembly of the viral particle discharged by the host cell. The NS3/4A protease, NS5A protein, and NS5B polymerase are inhibited by direct-acting antivirals (Salam and Akimitsu, 2013). Indole constitutes one of the most essential structural patterns in drug development and is considered a “privileged scaffold,” a term coined by Evans et al. (1988) to describe scaffolds that can serve as ligands for a variety of receptors. Researchers are working diligently to enhance the antiviral potency of novel indole derivatives as indole belongs to a class of alluring pharmacological substances. Indole scaffolds have been discovered to possess antimicrobial properties, antimalarial activities, and anti-tumor activity. Antiviral medications containing indole are developed to treat viral infections (Dorababu, 2020). A large number of researchers work around the clock to uncover antiviral drugs. Figure 1 demonstrates several compounds with anti-HCV and anti-HIV activity. Among them, compound I showed potent anti-HIV activity (IC_50_ = 1.4 μM) (Dorababu, 2020). In addition, 5,6-dihydroxyindole carboxamide derivative II displayed strong anti-HIV-1 integrase activity (IC_50_ = 1.4 μM). The *in vitro* IC_50_ value of delavirdine for HIV-1 averages 0.26 µM (Dueweke et al., 1993). Furthermore, indole derivatives IV and V displayed high anti-HCV activity, with EC_50_ values of 1.16 μM and 0.6 μM, respectively (Dorababu, 2020).

**FIGURE 2:**
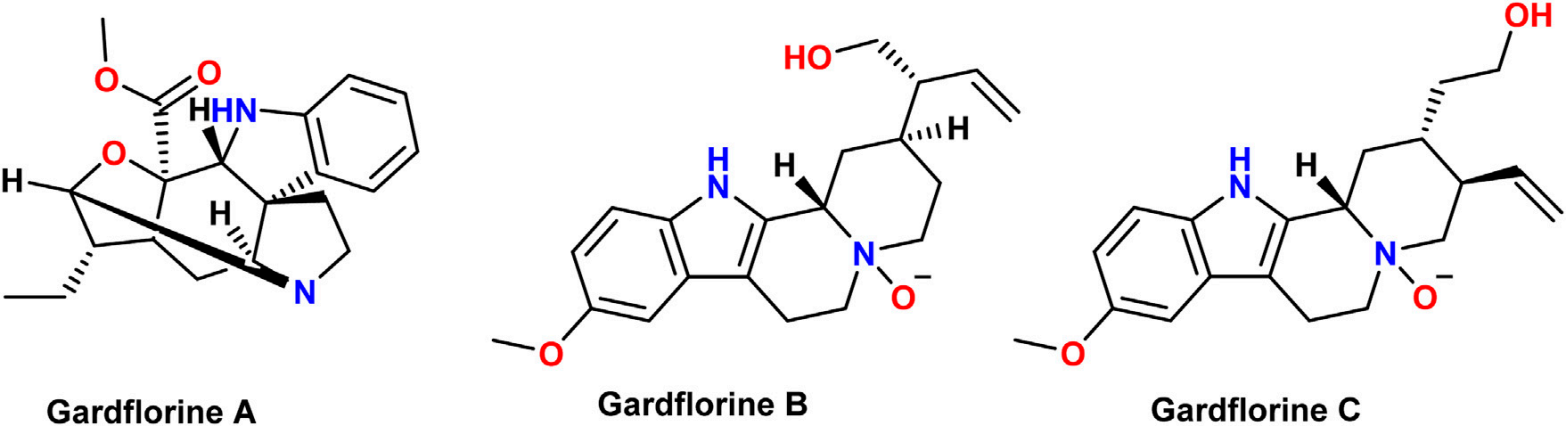
Structures of gardflorine **(A–C)**.

Many species of plants, animals, and marine organisms contain indole derivatives. The indole core is present in many physiologically active natural compounds (Zhang et al., 2015). *Gardneria multiflora* leaves were found to contain the monoterpenoid indole alkaloids gardflorine A, gardflorine B, and gardflorine C (Figure 2) (Zhang et al., 2021; Zhong et al., 2014). *Gardneria multiflora Makino*, a member of the Loganiaceae family, is mostly found in the southwestern region of China, and its stems have been used to cure food poisoning, snake bites, blisters, macula, dermatitis, herpes, and musculoskeletal pain (Yang et al., 2018). Gardflorine A displayed significant vasorelaxant activity, whereas gardflorine B (2) and gardflorine C inhibited AChE activity (Zhang et al., 2021).

This paper compiles and evaluates the antiviral activity of natural indoles, gardflorine **A–C**, using multi-target molecular docking studies against HIV and HCV, constant inhibition calculations (Ki), ADMET studies, frontier molecular orbital (FMO), natural bond orbital (NBO), and density functional theory (DFT) calculations.

## 2 Methodology

### 2.1 ADME studies

SwissADME was utilized to predict the ADME properties of each compound (last accessed on 20 February 2023, at http://www.swissadme.ch/index.php) (Daina et al., 2017).

### 2.2 Multiple target predictions

The LigTMap web server (https://github.com/ShirleyWISiu/LigTMap, last accessed 10 January 2023) (Shaikh et al., 2021) was used to perform multiple predictions for HCV and HIV targets with the tested compounds, gardflorine A–C, and the reference drugs.

#### 2.2.1 Inverse molecular docking

The crystal structures of the target enzymes were obtained from the Protein Data Bank (PDB). For the docking activities, Autodock Vina was utilized, which requires both the receptor and ligands to be in pdbqt extension. Before docking, M.G.L instruments were necessary to prepare the two enzymes (Trott and Olson, 2009). The docking findings were visualized using the Discovery Studio 4.5 visualizer (Dassault Systemes, 2023).

### 2.3 Inhibition constant (Ki value)

The binding energy was used to calculate the inhibition constant (Ki value) using the equation (ki = ^10 [Binding Energy/1.366]^) (Edwards et al., 2010).

### 2.4 Quantum chemical studies

The DFT calculations (Zhong et al., 2014) were done using ChemCompute Lab servers (Yang et al., 2018). The Becke, 3-parameter, Lee–Yang–Parr (B3LYP) level (Becke, 1993) with the 6–311++G (d,p) basis set (Papajak et al., 2011) has been utilized to optimize the molecular structure of the examined molecules, FMOs, and the molecular electrostatic potential (MEP). Various calculations were performed to determine the values of E_HOMO_, E_LUMO_, gap energy (∆E_gap_), ionization potential (I), electron affinity (A), electronegativity (χ), electronic chemical potential (μ), electrophilicity index (ω), global hardness (η), and global softness (S) calculated as outlined in the literature (Ismael et al., 2018) and then used to analyze the electronic features.

## 3 Results and discussions

### 3.1 ADMET

**FIGURE 3:**
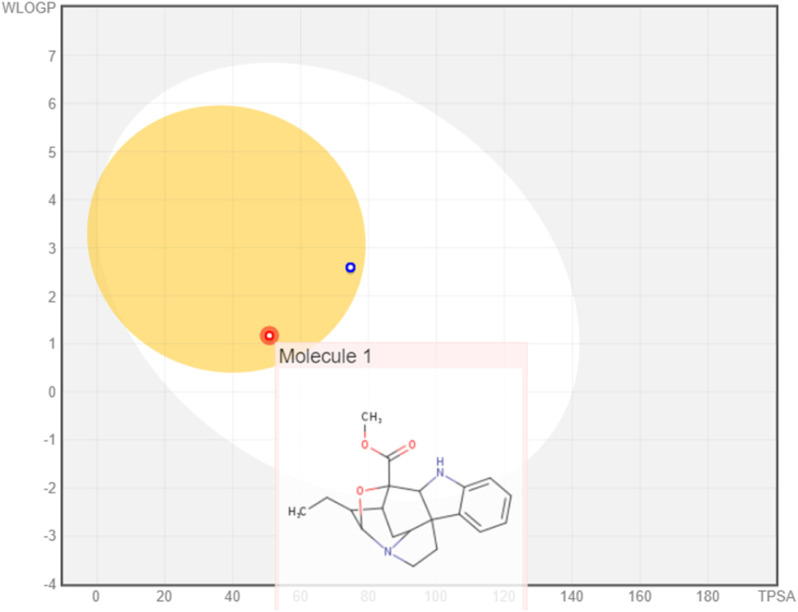
Boiled egg model of gardflorine A–C.

The boiled egg model revealed the ability of the three monoterpenoid indoles to penetrate the BBB; however, gardflorine A was shown to be a non-substrate to P-glycoprotein like B and C derivatives as shown in Figure 3 (Daina and Zoete, 2016; Şahin and Dege, 2021).

**FIGURE 4:**
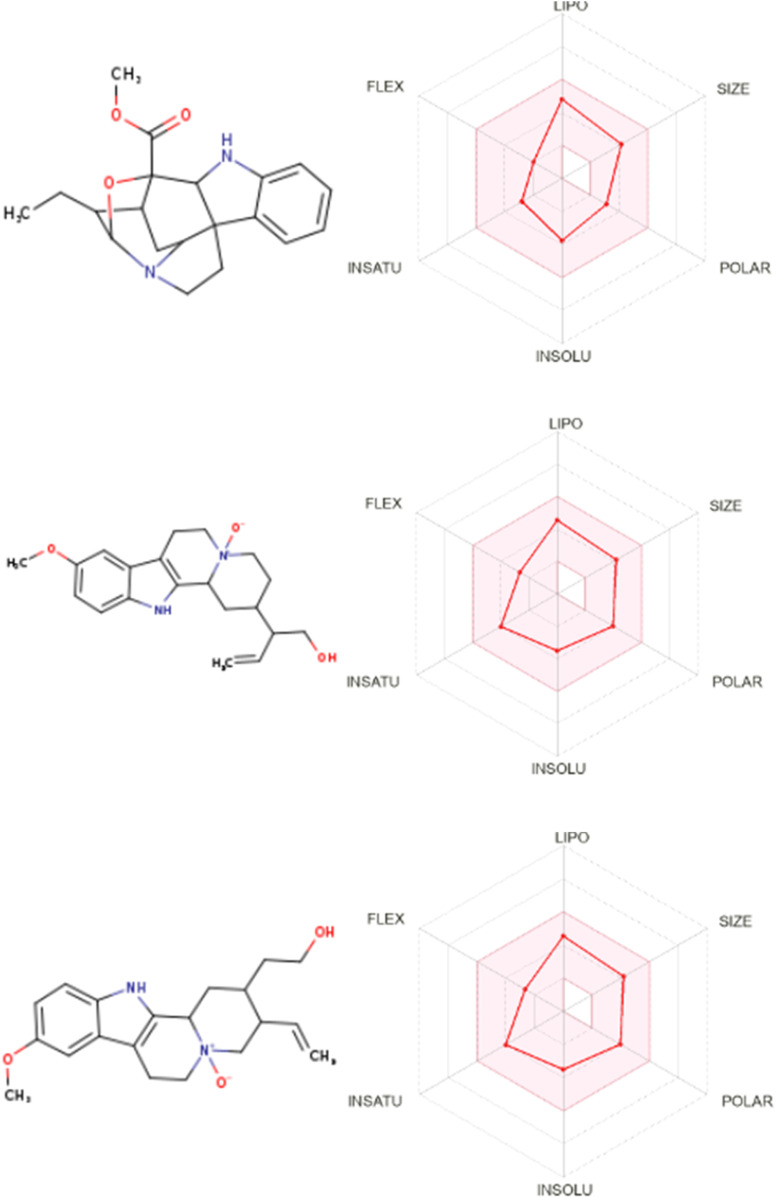
Bioavailability radar for gardflorine A–C.

Bioavailability radar charts for the tested compounds showed their good potential for bioavailability, as shown in Figure 4.

Table 1 illustrates the pharmacokinetic profile of gardflorine A–C, all revealed the ability to penetrate the BBB, and all also showed a high opportunity to be absorbed from the GIT. Gardflorine A only showed to be a non-substrate for P-glycoprotein. All are lead-like molecules with no violation of the Lipinski rule of 5.

**TABLE 1:** Pharmacokinetic profile for gardflorine A-C.

Item	Gardflorine-A	Gardflorine-B	Gardflorine-C
Formula	C_20_H_24_N_2_O_3_	C_20_H_26_N_2_O_3_	C_20_H_26_N_2_O_3_
Log *P*_o/w_ (iLOGP)	3.26	1.94	1.81
GI absorption	High	High	High
BBB permeant	Yes	Yes	Yes
P-gp substrate	No	Yes	Yes
Lipinski	Yes; 0 violation	Yes; 0 violation	Yes; 0 violation
Leadlikeness	Yes	Yes	Yes

### 3.2 Multiple targets of gardflorine A–C against HCV and HIV

**FIGURE 5:**
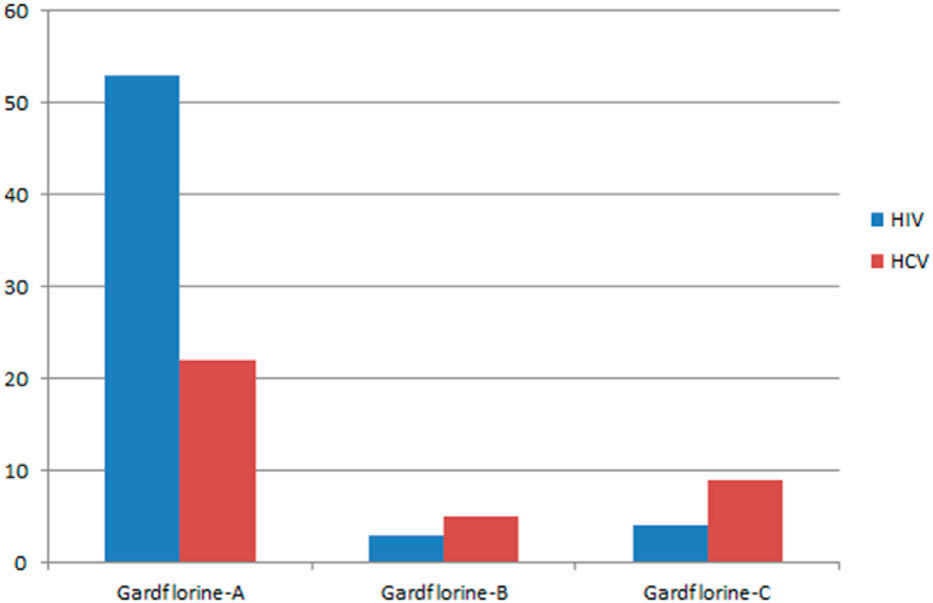
Bar chart illustrating a number of predicted targets of gardflorine A–C; Y-axis (no. Of targets), and *X*-axis (monoterpenoid indole).

**FIGURE 6:**
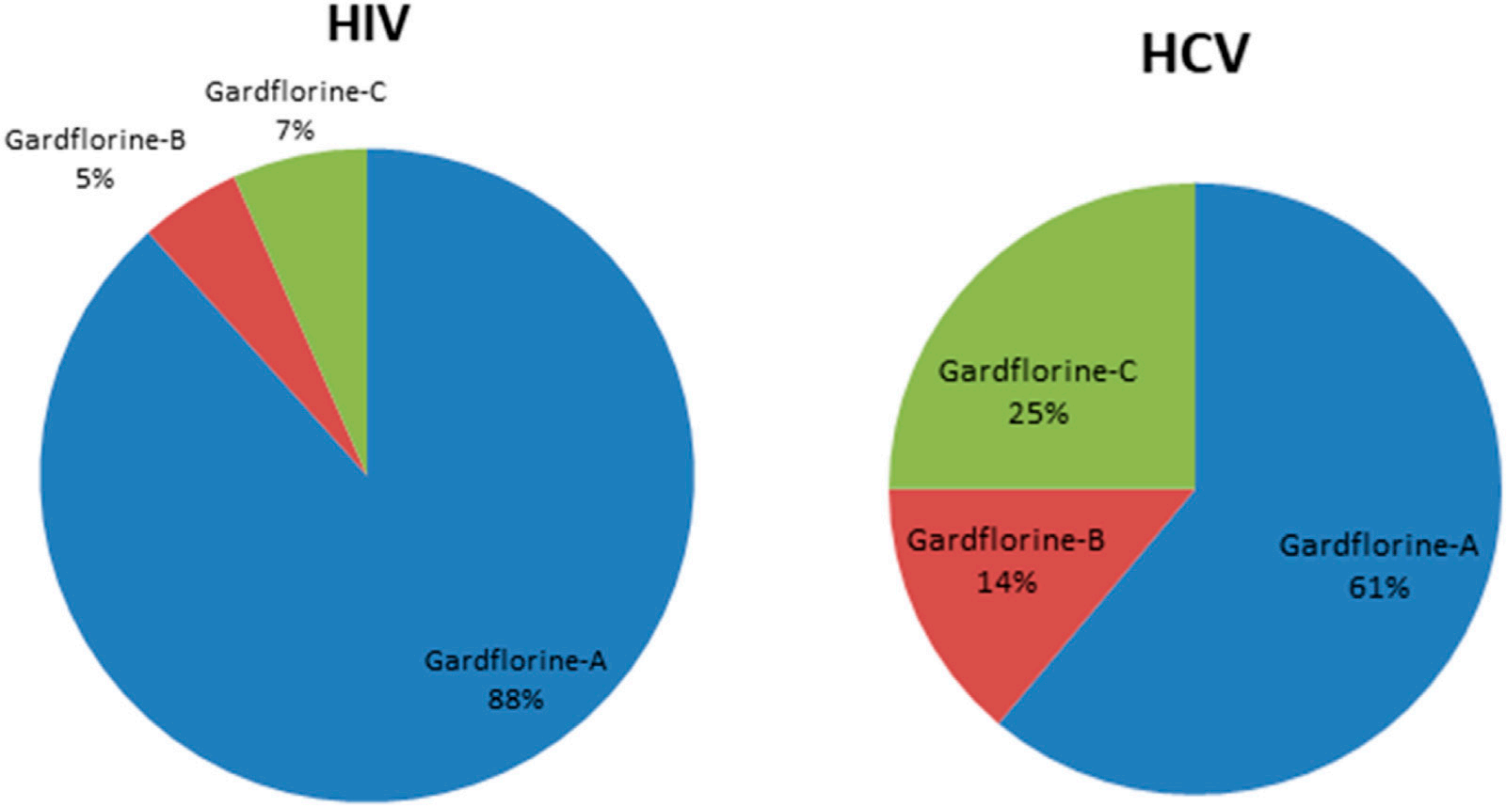
Pie chart illustrating percentage distribution of the predicted targets in HIV and HCV for gardflorine A–C.

Figures 5, 6 show the target distribution; gardflorine A can target more than 50 targets in HIV and more than 20 targets in HCV. Gardflorine B and C showed more selectivity toward HCV proteins than HIV targets compared to gardflorine A.

### 3.3 Molecular docking and inhibition constant (Ki value)

Docking studies were performed for gardflorine A–C and delavirdine against protein targets in HIV and HCV. Delavirdine displayed the ability to target 29 HCV-affecting proteins and 14 HIV-affecting proteins. Gardflorine A revealed the ability to target 53 proteins (Figures 5, 6) that affect HIV and 21 proteins that affect HCV (Figures 5, 6). Gardflorine B and C revealed the ability to target three and four proteins that affect HIV, respectively, and five and nine proteins that affect HCV, respectively. All proteins are listed in Supplementary Tables 1–4 with their pdb ID codes, ligand names, similarity and docking scores, and inhibition constant (Ki value).

The docking methodology was rigorously validated through re-docking and superimposition of the native ligands originally co-crystallized within the active sites of target proteins (pdb ID: 2PK5, 2PK6, 3HKW, 3I0R, 3NF6, 3OK9, 3QO9, 3UPI, 3VQS, 4NWK, 4ZIP, 5EQQ, 5ETX, and 5KGW). During the validation, the binding pose of this co-crystallized ligand was accurately reproduced, ensuring that the docking protocol was reliable. The superimposition of the re-docked ligand with the native ligand demonstrated a high degree of alignment, confirming that the docking procedure could precisely mimic the original ligand’s interactions within the active site. This successful validation, depicted in Fig. S. X (supplementary data), underscores the robustness and accuracy of the docking protocol, making it suitable for predicting the binding modes of other compounds in subsequent studies.

### 3.4 Molecular docking studies against HCV proteins

#### 3.4.1 RNA-directed RNA polymerase

**FIGURE 7:**
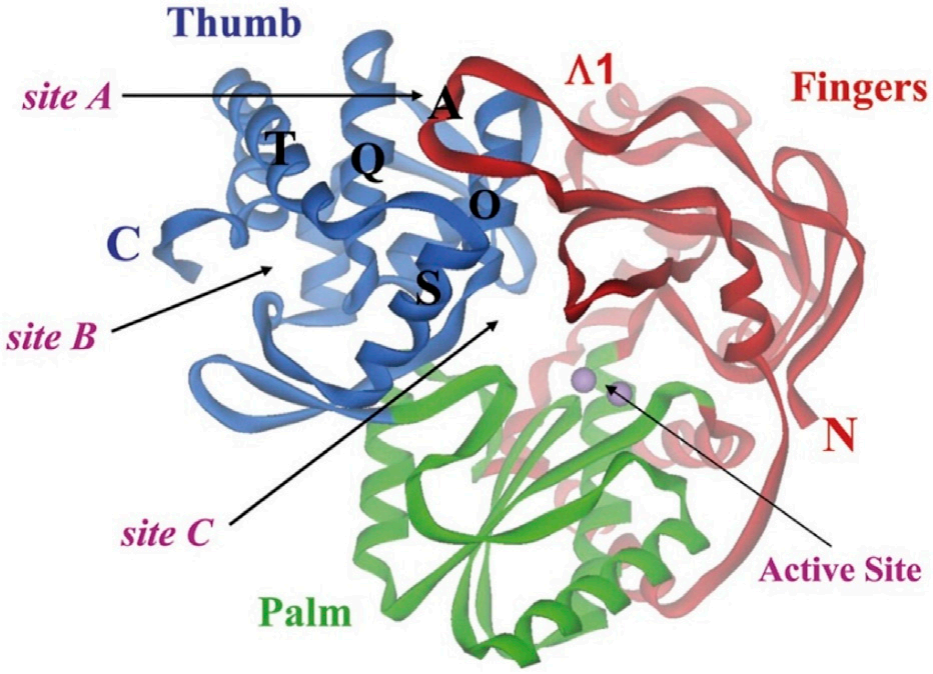
Binding sites on HCV NS5B polymerase. The three locations are confined inside the helices O, Q, S, and T. Each of the sites A and B is situated on the surface of the thumb domain. Site C is near the enzyme active site (Rydberg et al., 2009).

**FIGURE 8:**
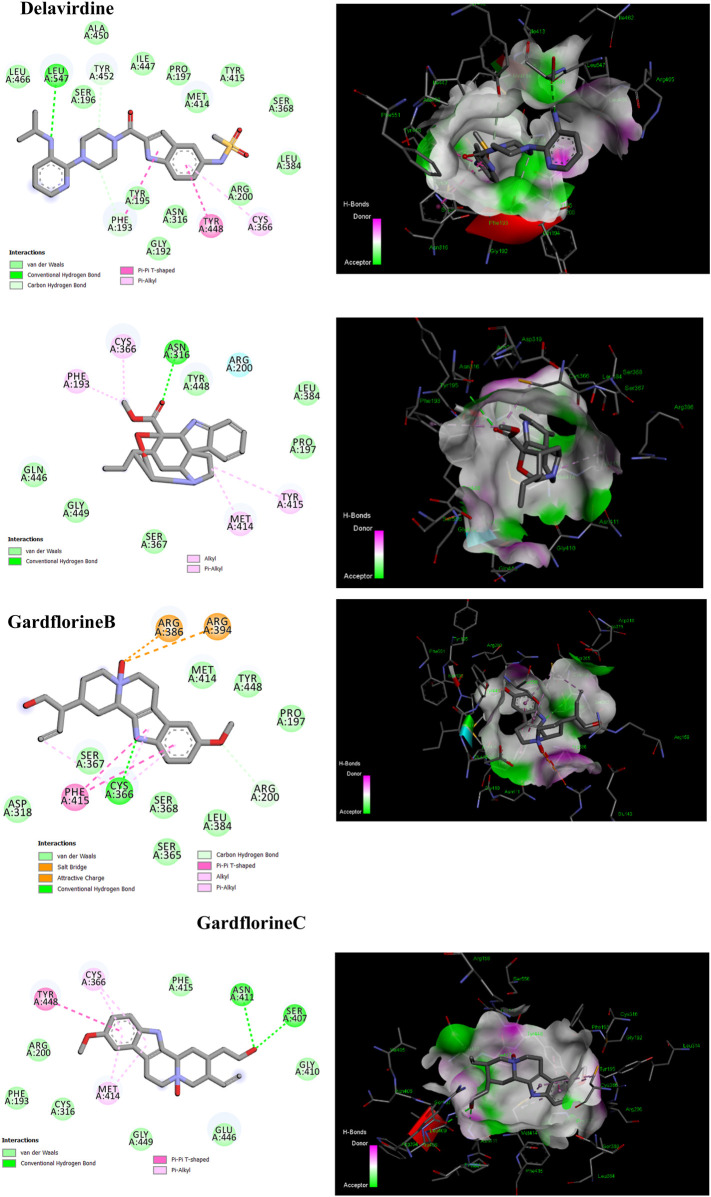
2D and 3D representation of the predicted binding mode for compounds delavirdine (PDB ID: 3vqs), gardflorine A (PDB ID: 3upi), gardflorine B (PDB ID: 3hkw), and gardflorine C (PDB ID: 3hkw) against HCV RNA-directed RNA polymerase.

One of the primary focuses of medication research and development is a viral protein called HCV NS5B RNA polymerase, which plays a crucial role in the replication of the HCV gene (Shaw et al., 2009). Similar to other members of the Pol I family, the HCV NS5B crystal structure reveals an overall subdomain architecture with a deep active site cavity at the top of the “palm” subdomain, sealed at its base by a distinctive b-loop (Ikegashira et al., 2006). Moreover, an unexpected interaction was found between the tip of the “fingers” subdomain and the “thumb” subdomain, which serves to ring the hypothesized nucleoside triphosphate substrate entrance trajectory (Tedesco et al., 2006). Sequence variation analysis reveals that residues lining the active site cavity (“palm site”) are more conserved than in locations such as the “thumb site (Figure 7). This renders the palm site an intriguing target for the inhibition of the viral polymerase, although not all residues surrounding this site are entirely conserved. Non-nucleoside inhibitors that bind to the palm, thumb, and finger-loop subdomains are effective in clinical trials (Velázquez et al., 2012). The X-ray structure of NS5B verifies that the ligand interacts with Cys366, Met414, Leu384, and Tyr415 in the “palm site” of the active site cavity of the apoprotein (Ando et al., 2012). Via its indole moiety, delavirdine displayed three Pi interactions with residues Try448, Phe193, and Cys366. In addition, it formed three hydrogen bonds with Leu547, Phe193, and Try452 residues, with two additional interactions involving Phe193 and Try452 (Figure 8; Supplementary Table 5). The natural indole compounds gardflorine A, gardflorine B, and gardflorine C with docking scores of −7.35, −7.64, and −7.56 kcal/mol (Table 2; Supplementary Table 5), respectively, are also docked to the same binding site of the RNA-polymerase enzyme as delavirdine. The three natural indole compounds showed pi-alkyl interactions with the crucial amino acid Cys366. Gardflorine A formed one hydrogen bond interaction with Asn316, in addition to four hydrophobic interactions. The indole moiety of gardflorine B and C shared more than four interactions with various amino acids in the active site cavity (palm site) (Figure 8; Supplementary Table 5). In the case of gardflorine B, it interacted with Cys366 and Phe415, and gardflorine C interacted with Cys 366, Tyr 448, and Met414. The inhibition constant (Ki) values of delavirdine and gardflorine A, B, and C were 1.85, 4.11, 2.52, and 2.92 µM, respectively.

**TABLE 2:** Protein targets with the highest docking score in HIV and HCV, Pdb IDs, ligand names, ligand similarity and docking scores of delavirdine, monoterpenoid indole gardflorine A, B, and C, and calculated inhibition constant values.

Compound	PDB	Target class	Target name	Ligand name	Ligand similarity score	PSO Vina2 docking score (kcal/mol) ▾	Predicted Ki (µmol)
Delavirdine	3vqs	HCV	RNA polymerase	JT1	0.442	−7.83	1.85
	5etx	HCV	NS3/A4 protease	5RS	0.423	−6.691	12.64
	4zip	HIV	Protease	G64	0.406	−7.804	1.93
	3i0r	HIV	Reverse transcriptase	RT3	0.412	−8.157	1.06
Gardflorine A	3upi	HCV	RNA polymerase	0C2	0.405	−7.357	4.11
	5etx	HCV	NS3/A4 protease	5RS	0.426	−6.231	27.44
	3ok9	HIV	Protease	G52	0.407	−9.722	0.076
	3qo9	HIV	Reverse transcriptase	QO9	0.409	−8.389	0.72
	3nf6	HIV	Integrase	IMV	0.401	−6.079	35.46
Gardflorine B	3hkw	HCV	RNA polymerase	IX6	0.408	−7.647	2.52
	4nwk	HCV	NS3/A4 protease	2R8	0.401	−6.156	31.14
	2pk6	HIV	Protease	O33	0.401	−5.899	48.03
	5kgw	HIV	Integrase	7SK	0.404	−4.827	292.63
Gardflorine C	3hkw	HCV	RNA polymerase	IX6	0.419	−7.56	2.92
	5eqq	HCV	NS3 protease	5RS	0.402	−7.03	7.13
	2pk5	HIV	Protease	075	0.405	−7.447	3.53
	5kgw	HIV	Integrase	7SK	0.404	−4.598	430.4

#### 3.4.2 NS3/A4 protease

**FIGURE 9:**
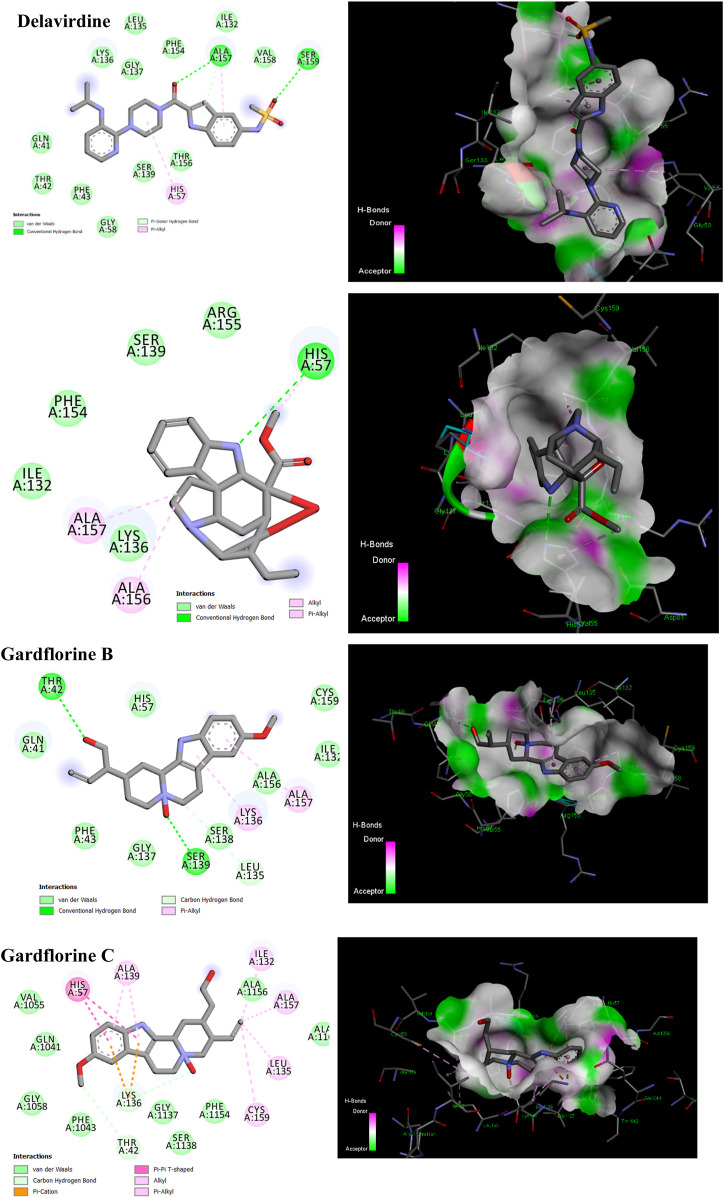
2D and 3D representation of predicted binding mode for compounds delavirdine (PDB ID: 5etx), gardflorine A (PDB ID: 5etx), gardflorine B (PDB ID: 4nwk), and gardflorine C (PDB ID: 5eqq) against HCV N53 protease.

Four known locations along the virally encoded polyprotein are cleaved by the HCV NS3/4A protease, a chymotrypsin-like serine protease that is a prime therapeutic target (Lemke et al., 2011). Pharmaceutical companies have made substantial investments in the development of NS3/4A protease inhibitors (Ikegashira et al., 2006). Most inhibitors interact with the Arg155, Ala 157, and Ser159 residues of the protease backbone. Additionally, the catalytic amino acids serine 139 and His 57 are essential for proper binding (Scola et al., 2014). Delavirdine formed three hydrogen bonds with Ser159 and Ala157, and docking scores of −6.69 kcal/mol were obtained, along with hydrophobic interactions with His57 and Ala157 (Figure 9; Table 2; Supplementary Table 6). Gardflorine A and C bind to the catalytic His 57 residue and the essential Ala157 amino acid (Figure 9; Table 2; Supplementary Table 6). Gardflorine A displayed one H-bond with the catalytic residue His 57 (3.20 Å) and three alkyl bonds with Ala156, Ala157, and His57 with a docking score equal to −6.23 kcal/mol. In the case of gardflorine B, the catalytic amino acid serine 139 contributes two hydrogen bonds. Moreover, gardflorine B showed another two H-bonds with Leu 135 and Thr 42 and two pi–alkyl bonds with Lys136 and Ala157 with a docking score equal to −6.15 kcal/mol (Figure 9; Table 2; Supplementary Table 6). Gardflorine C interacts through five types of interactions and forms 14 bonds with essential residues, including Lys136, Thr42, Lys 136, His 57, Ala157, Ile132, Cys159, and Ala139 (Figure 9; Table 2; Supplementary Table 6). Moreover, gardflorine C had the lowest inhibition constant (Ki) of 7.13 µM.

### 3.5 Molecular docking studies against HIV proteins

#### 3.5.1 Protease

**FIGURE 10:**
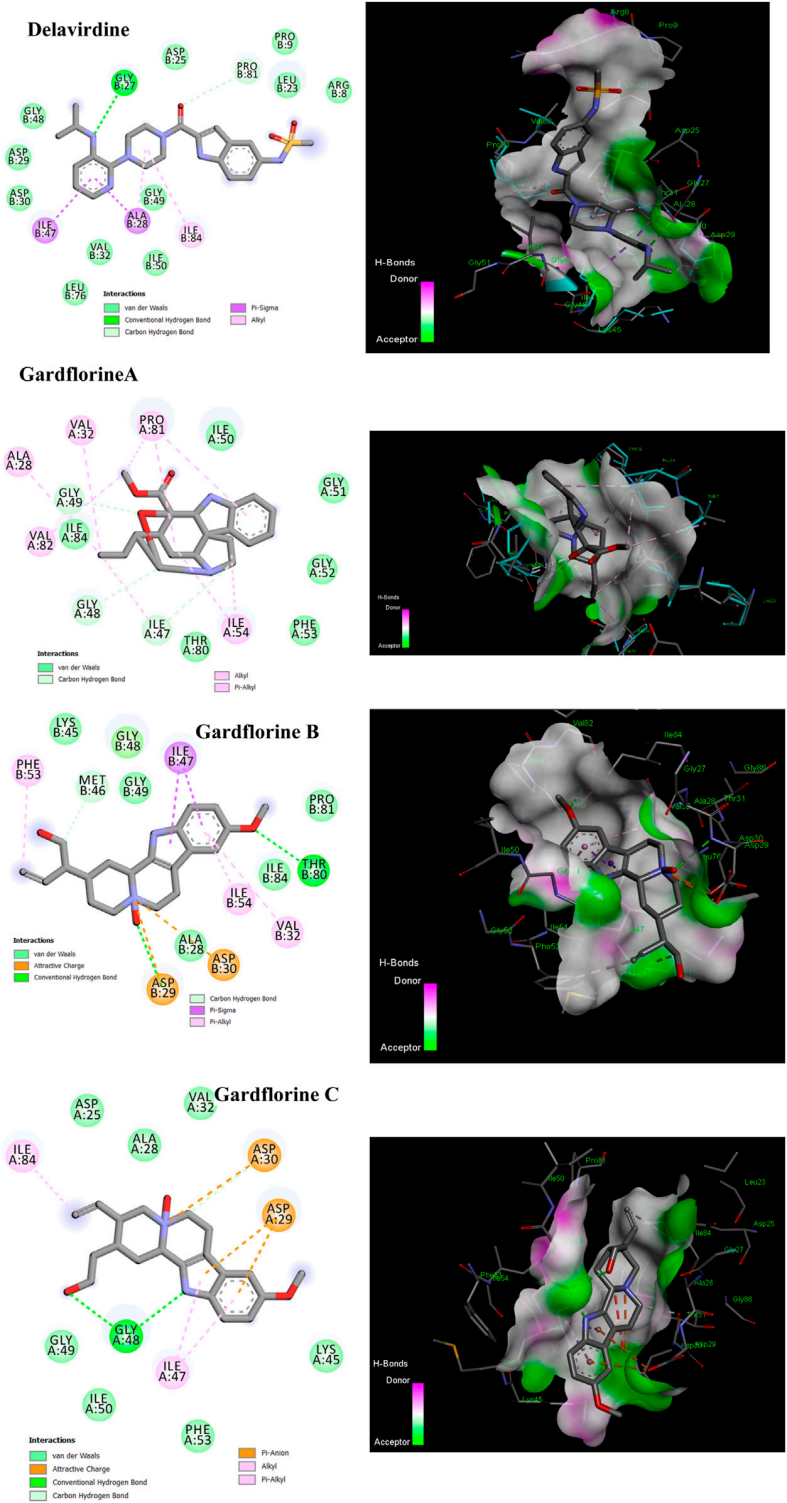
2D and 3D representations of the predicted binding mode for compounds delavirdine (PDB ID: 4zip), gardflorine A (PDB ID: 3ok9), gardflorine B (PDB ID: 2pk6), and gardflorine C (PDB ID: 2pk5) against HCV N53 protease.

As a dimer, HIV-1 protease (PR) is catalytically active, and the catalytic Asp25 residues from both subunits interact strongly at the subunit interface (Lafont et al., 2007). The binding site contains the residues Ala28, Asp29, Asp30, Met46, Val82, Val32, Ile47, and Ile84 (Tie et al., 2004). Some inhibitors bind via van der Waals forces with the protease residues Leu23, Gly49, Ile50, Pro81, Val82, and Ile84 from both subunits (Zhang et al., 2013). Delavirdine had fewer hydrogen bond interactions with the protease residues Gly27 and Pro81, two pi–sigma bonds with Ala 28 and Ile47, and two alkyl bonds with Ala28 and Ile84; its docking score was −7.80 kcal/mol (Figure 10; Table 2 and Table S7). Gardflorine A–C anchored correctly in the binding site of the HIV-protease enzyme. Gardflorine A showed the highest docking score of −9.72 kcal/mol and a higher number of hydrogen bonds than gardflorine B and C (Figure 10; Table 2, and Supplementary Table 7). Gardflorine A revealed four hydrogen bonds with Gly49 and Ile47, along with nine alkyl interactions with Ala28, Pro81, Ile54, Val32, Ile47, and Val82. Gardflorine B exhibited the lowest docking score of – 5.89. Delavirdine and gardflorine A, B, and C exhibited respective inhibition constants (Ki) of 1.93, 0.076, 48.03, and 3.53 µM.

#### 3.5.2 Reverse transcriptase

**FIGURE 11:**
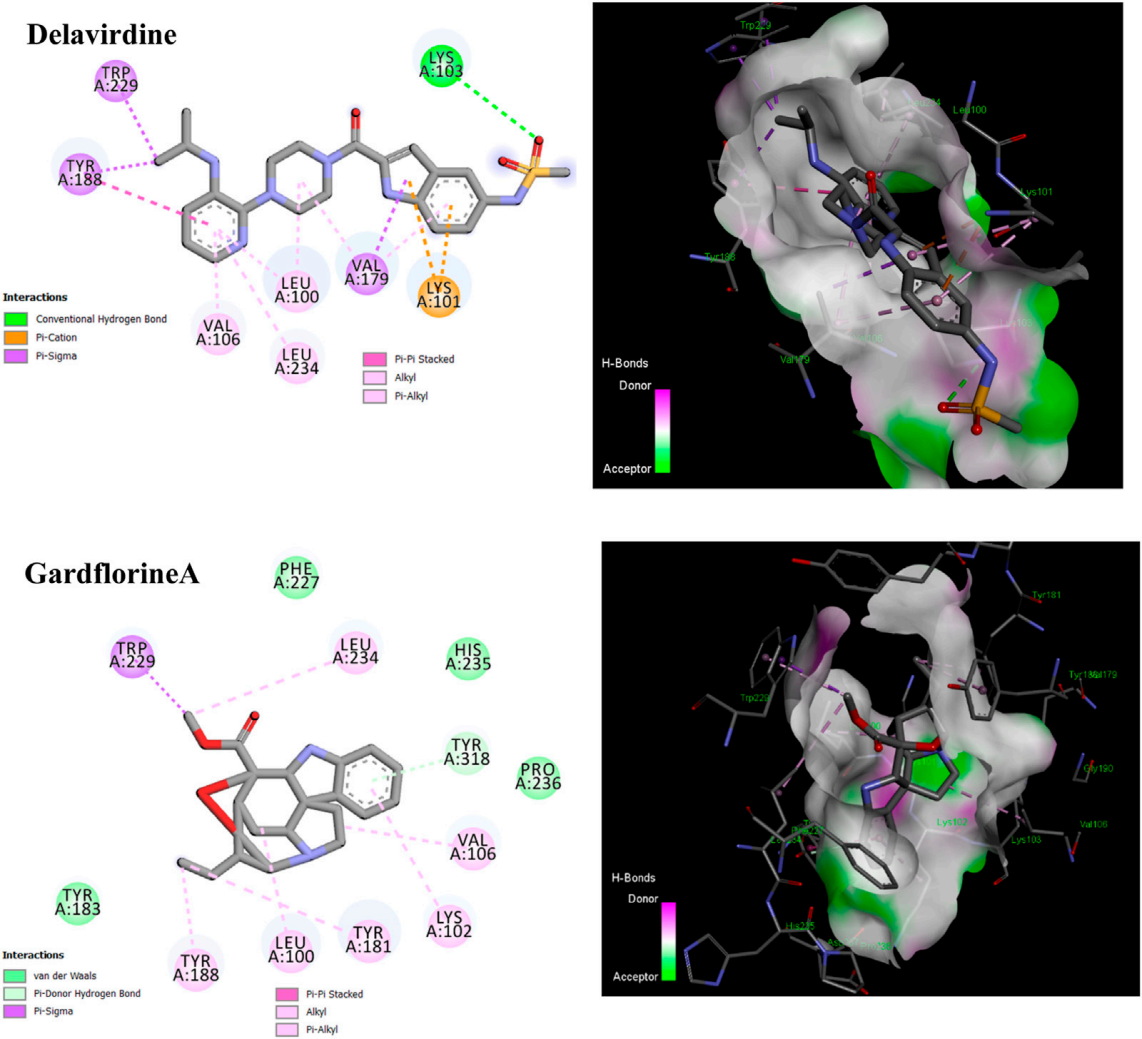
2D and 3D representations of the predicted binding mode for compounds delavirdine (PDB ID: 3i0r) and gardflorine A (PDB ID: 3qo9) against HCV RT.

p66 (66 kDa) and p51 (51 kDa) subunits form a heterodimer to form HIV-1 RT. Three catalytic trio aspartate residues (Asp110, Asp185, and Asp186) are necessary for DNA polymerization (Parrish et al., 2013). Non-nucleoside reverse transcriptase inhibitors (NNRTIs) are essential components of HIV-1 infection-treating multidrug regimens known as HAART (highly active antiretroviral treatment). It was reported that the drug’s interaction with Trp229 and Lys103 results in extraordinary effectiveness (Su et al., 2009; Das et al., 2011). According to our investigation, neither gardflorine B nor gardflorine C was an HIV-reverse transcriptase target. Gardflorine A docked in the same manner as delavirdine at the active site. In addition, gardflorine A showed higher docking scores than delavirdine, −8.38 *versus* −8.15 kcal/mol. Delavirdine displayed three hydrogen bonds and thirteen hydrophobic interactions. Gardflorine A exhibited hydrogen bond interactions with Try 318; a pi bond with Try229, Tyr 318, Try 181, Try 188, Trp 229, and Lys102; and a pi-stacking interaction with Try 181, Try 188, and Try 188. Moreover, three alkyl linkages occurred between gardflorine A and Leu100, Val 106, and Leu 234 (Figure 11; Table 2, and Supplementary Table 8). Delavirdine and gardflorine A have respective inhibition constants (Ki) of 1.06 and 0.72 µM.

#### 3.5.3 Integrase

**FIGURE 12:**
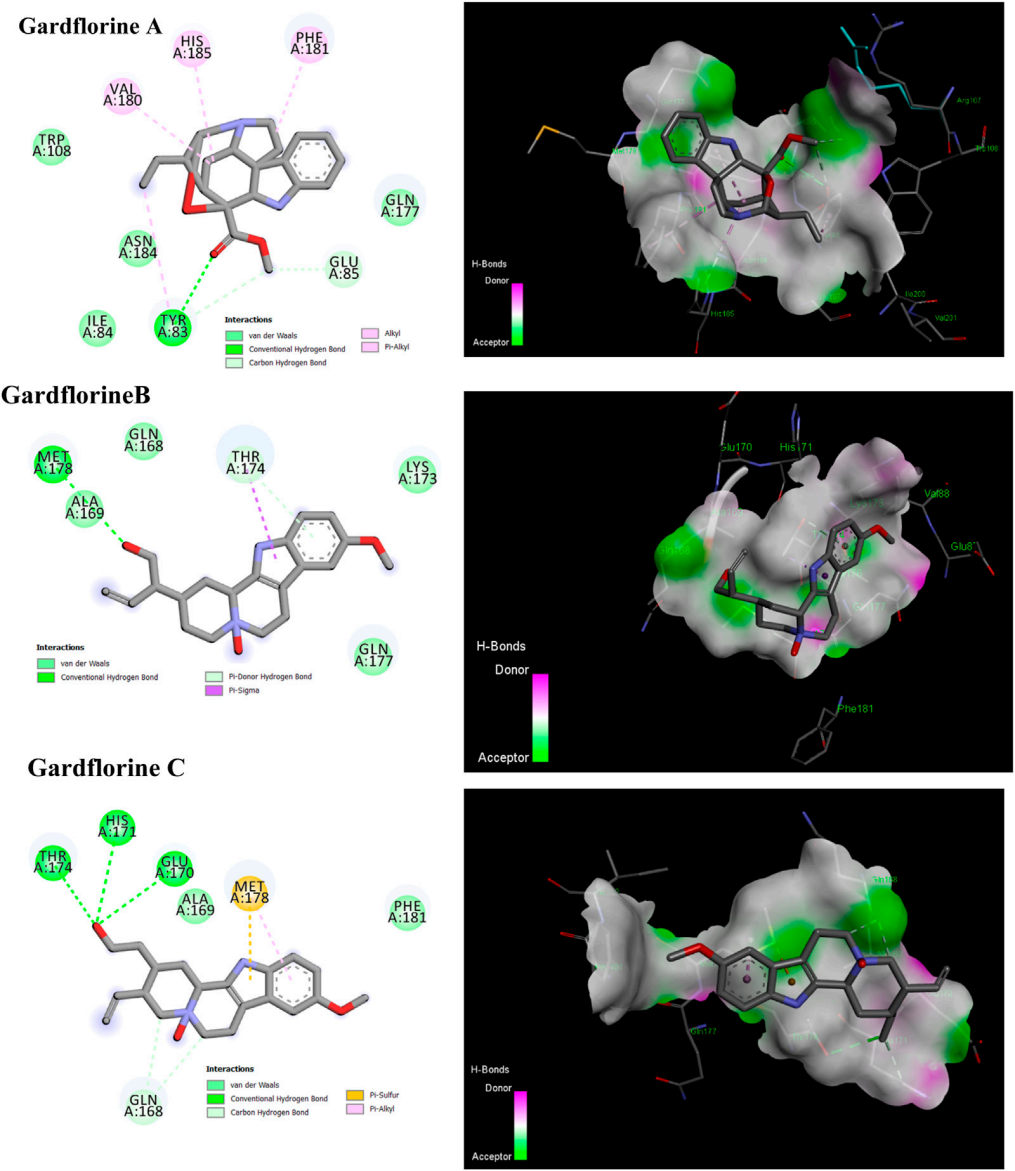
2D and 3D representations of the predicted binding mode for compounds gardflorine A (PDB ID: 3nf6) and gardflorine B and C (PDB ID: 5kgw) against HCV-Integrase.

By facilitating the insertion of viral DNA into the host genome, HIV-1 integrase (IN) plays a vital role in viral replication. The entire process is mediated by the well-ordered formation of a stable synaptic complex (SSC) through the multimerization of HIV IN into a tetramer on viral DNA. Given the significance of HIV-1 IN for viral infection, there has been considerable interest in the development of drugs capable of inhibiting IN activity (Rhodes et al., 2011). The enzymatic activity of integrase requires two magnesium ions to be coordinated by a DDE motif (Asp64, Asp116, and Glu152) at the active site of integrase, with the position of Glu152 modified by the mobile loop (residues Gly140 to Gly149), suggesting that the loop plays a role in metal positioning (Gupta et al., 2014). Gardflorine A has the lowest binding energy, at −6.07 kcal/mol; it interacts with Tyr 83 and Glu85 by forming three H-bonds and alkyl bonds with Val180, Tyr 83, Phe 181, and His 185. Gardflorine B and C have docking scores of −4.82 and −4.59, respectively, and they interact with the complementary pocket residues Thr 174 Met 178. Gardflorine C exhibited six hydrogen bonds with Glu 170, His 171, Thr174, and Gln168, along with pi–sulfur and pi–alkyl bonds with Met 178 (Figure 12; Table 2; Supplementary Table 9). The inhibition constants (Ki) for gardflorine A, B, and C were 35.46, 292.63, and 430.4 µM, respectively.

## 4 Quantum chemical studies

### 4.1 FMO analysis and chemical reactivity

**FIGURE 13:**
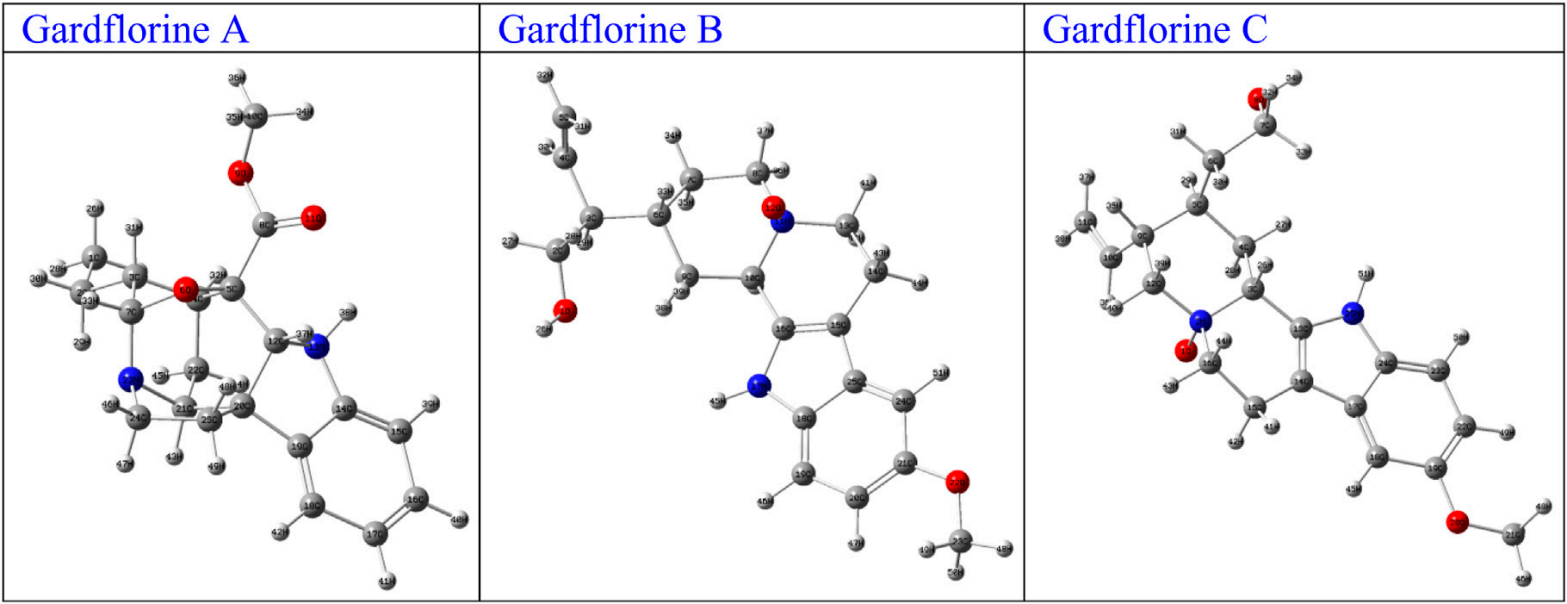
Optimized structures of gardflorine (A–C) with the scheme of atom numbering obtained by B3LYP/6–311++G (d,p) in the gas phase.

The optimized structures of the title compounds are shown in Figure 13. The most significant concept for researchers that provides data on chemical reactivities is the FMO (Ismael et al., 2021a). FMOs refer to the energies of a compound’s lowest unoccupied molecular orbital (LUMO) and highest occupied molecular orbital (HOMO). These orbitals control how the molecule interacts with other species. HOMO stands for the ability to give an electron, whereas LUMO stands for the ability to take an electron.

For A, B, and C, respectively, the calculated HOMO energies were −5.52, −4.81, and −5.16 eV, while the corresponding LUMO energies were −0.81, −0.65, and −0.74 eV. The molecule’s chemical stability is described by the gap energy (ΔE_gap_) (Ismael et al., 2021b). The Egap values of A, B, and C were determined to be 4.71, 4.16, and 4.43 eV, respectively. Normally, when the ΔE_gap_ is small, the molecule is highly polarizable and is associated with low kinetic stability and high chemical reactivity, and it is referred to as a soft molecule. In conclusion, the title molecule structure’s biological reactivity is demonstrated by the low value of ΔE_gap_ (Abdou et al., 2022).

**FIGURE 14:**
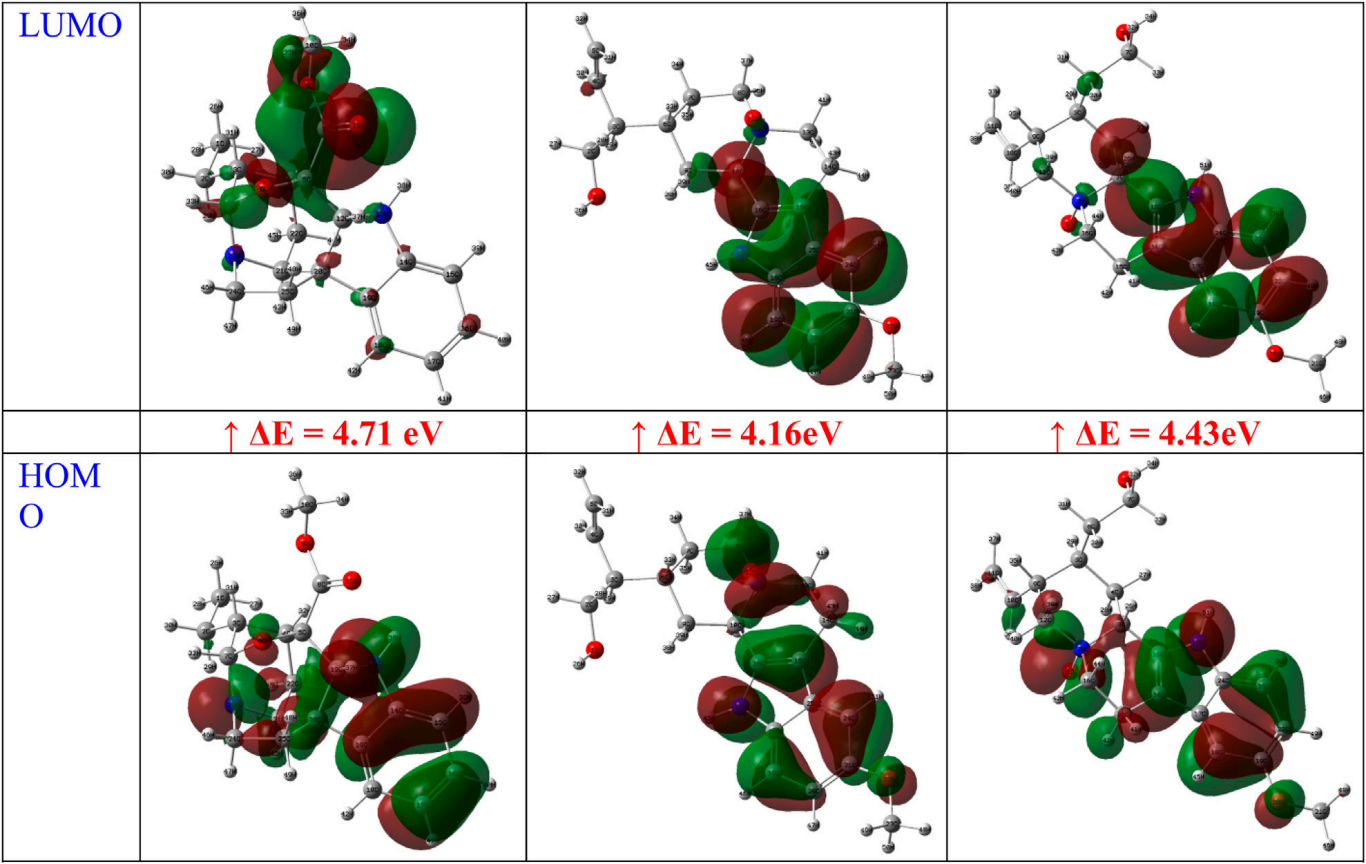
FMOs of gardflorine (A–C) at B3LYP/6–311++G (d,p) in the gas phase.

The HOMO and LUMO map’s incorporation in molecules A, B, and C are depicted in Figure 14. The molecular orbital wave function’s negative and positive phases are represented, respectively, by the green and red color distributions. These images show that the lowest unoccupied molecular orbitals, or LUMOs, and the highest occupied molecular orbitals, or HOMOs, are mostly concentrated across the whole molecule structure, as shown in Figure 14.

Understanding the connection between structural stability and global chemical reactivity relies on the knowledge of global reactivity parameters. The calculated values of the HOMO and LUMO energies, the gap energy (ΔE_gap_), the ionization potential (I), the electron affinity (A), the total energy of the optimized molecular structure (E_Total_), and some global reactivity properties like electronegativity (χ), the electronic chemical potential (μ), the electrophilicity index (ω), global hardness (η), and global softness (S) for the A, B, and C structures in the gas are tabulated in Table 3. The optimized geometries of the A, B, and C atoms have total energy values (E_Total_) of −30256.87, −30287.96, and −30260.86 e.V., respectively, as shown in Table 3, indicating high stability.

**TABLE 3:** Calculated chemical parameters.

	E_Total_	*µ*	*E*_*HOMO*_	*E*_*LUMO*_	*∆E*	*I*	*A*	*χ*	*CP*	*η*	*S*	*ω*
A	−30256.87	1.31	−5.52	−0.81	4.71	5.52	0.81	3.17	−3.17	2.35	0.21	2.13
B	−30287.96	4.18	−4.81	−0.65	4.16	4.81	0.65	2.73	−2.73	2.08	0.24	1.79
C	−30260.86	6.05	−5.16	−0.74	4.43	5.16	0.74	2.95	−2.95	2.21	0.23	1.97

As for the dipole moment (*µ*) value, it was discovered to be 1.31, 4.18, and 6.05 Debye, respectively, for the A, B, and C structures produced using the DFT technique in the gas phase. The ionization potential (I) values of the structures A, B, and C are lower than the average (5.52, 4.81, and 5.16 eV), which suggests that they have better electron donor properties. Additionally, it was determined that the values of A, B, and C’s global chemical hardness (η) and softness (S) were (2.35, 2.08, and 2.21 eV) and (0.21, 0.24, and 0.23 eV), respectively. One interpretation of the values is as an indicator of intramolecular charge transfer. Additionally, the low and high chemical hardness (η) and softness (S) values obtained show that the studied structure is a soft molecule.

The electrophilic index (ω) of the A, B, and C structures was determined to be 2.13, 1.79, and 1.97 eV, respectively. According to Domingo *et al.*’s classification of organic compounds, the title molecule structurally falls into the category of “high electrophiles” (>1.50 eV). The ability of an atom or set of atoms to draw electrons is quantified by the electronegativity (χ) descriptor. According to calculations, A, B, and C’s electronegativity and electronic chemical potential (CP) are, respectively, (3.17, 2.73, and 2.95) eV and (−3.17, −2.73, and −2.95) eV.

### 4.2 Molecular electrostatic potential

**FIGURE 15:**
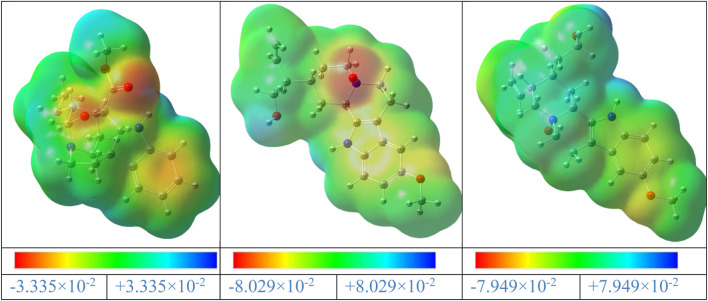
MEP maps of the gardflorine (A–C) molecules computed using the B3LYP/6–311++G (d,p) in the gas phase.

The MEP is a valuable tool for illustrating the electronic density in molecules, and it is used to identify places with surfaces that have both positive and negative electrostatic potentials by utilizing different colored dots (Domingo et al., 2002). On the other hand, the red, orange, or yellow negative sites (high electron density) represent the electrophilic assault, the green positive sites (low electron density) reflect the nucleophilic attack, and the blue positive sites (high electron density) represent the neutral regions. A, B, and C’s MEP surfaces were calculated using the B3LYP/6–311++G (d,p) (Gas) level of theory (Figure 15).

The negative areas of the A, B, and C molecules were located around the oxygen atoms, as shown in Figure 15. Additionally, the hydrogen atom linked to the nitrogen in the A, B, and C structures is the center of the positive areas, making it vulnerable to nucleophilic assault. Additionally, the areas with faint blue coloring represent weak interaction locations. Additionally, the places of the title compounds’ structures that are colored green display neutral areas with no potential.

### 4.3 Natural charge analysis

**FIGURE 16:**
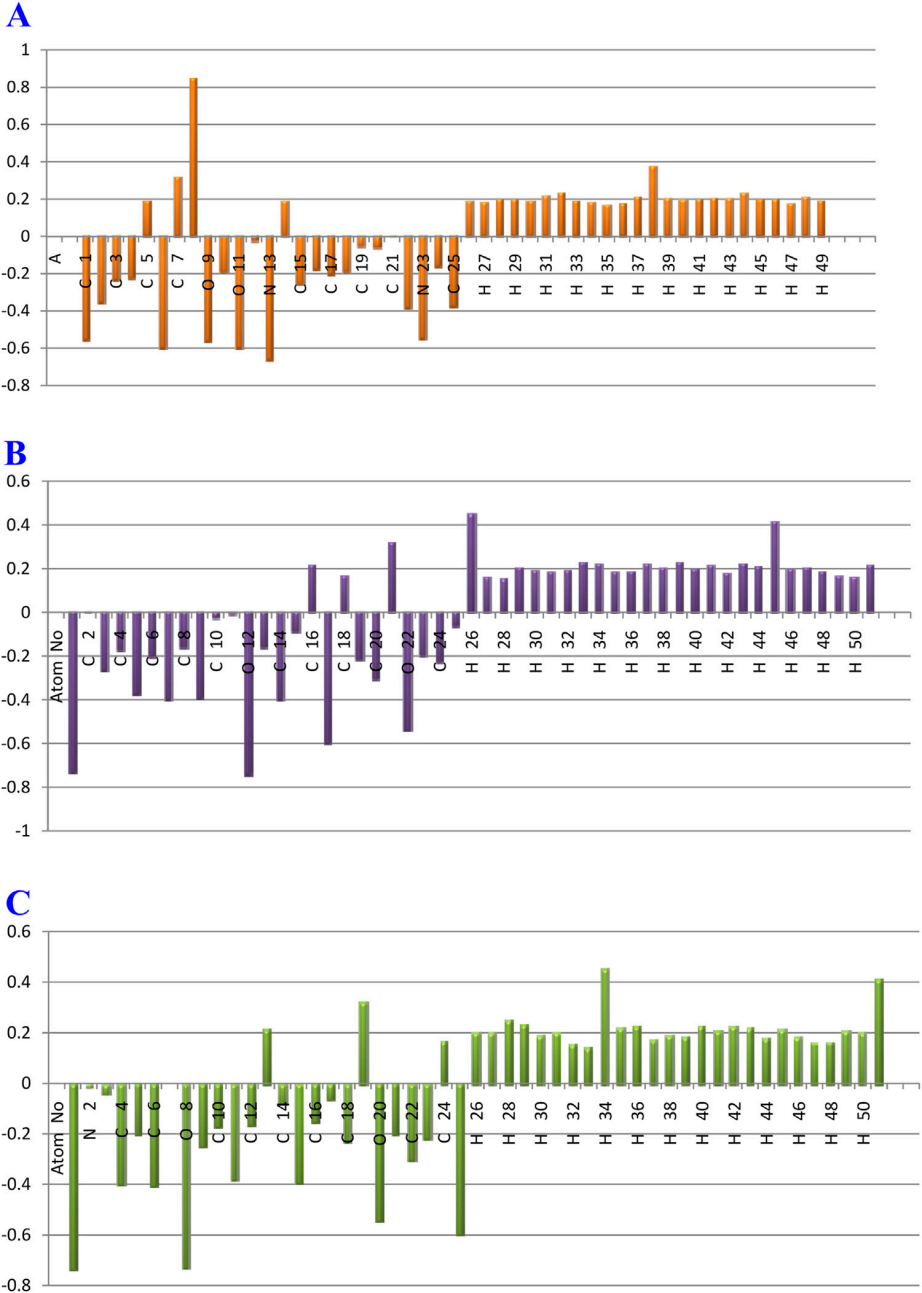
Plot of natural charge distribution of the gardflorine **(A–C)** molecules computed using the B3LYP/6–311++G (d,p) in the gas phase.

Because they represent the physicochemical characteristics of a molecule (the electronic structure, vibrational spectra, dipole moment, polarizability, and other molecular properties), atomic charges play a significant role in molecules (Shahab et al., 2020). The atomic charges of A, B, and C molecules in the current investigation were calculated using NBO analysis at the B3LYP/6–311++G (d,p) level of theory in the gaseous phase. The findings are presented in Supplementary Table 11, and the atoms are numbered in accordance with Figure 16.

Understanding electronegativity equalization and charge transfer in the chemical reactivity of the title molecule is made easier with the use of NBO analysis. The NBO analysis of the molecule reveals that carbon atoms in A, B, and C contain both positive and negative charges. Positive carbons are observed for carbon atoms coupled with the electron-withdrawing oxygen and nitrogen atoms, as illustrated in Supplementary Table 11 and Figure 16, including (C14, C5, C7, and C8), (C2, C18, C16, and C21) and (C7, C24, C13, and C19) atoms in the tile A, B, and C molecules, respectively. Moreover, the other carbon atoms, including carbon atoms (C22, C25, C2, C15, C3, C4, C17, C18, C10, C16, C24, C20, and C19), (C14, C7, C9, C5, C20, C3, C24, C19, C6, C23, C4, C8, C13, C15, C25, and C10) and (C6, C4, C15, C11, C22, C9, C18, C23, C5, C21, C10, C12, C16, C14, C17, and C3), have a negative charge in the tiled A, B, and C molecules, respectively.

Additionally, C8 (0.85006e), C21 (0.31877e), and C19 (0.32007e) atoms in the A, B, and C molecules, respectively, have the largest positive charges due to their connection to withdrawing oxygen atoms (O11, O9), (O22), and (O20), as opposed to other carbon atoms. The most negatively charged atoms in compounds A, B, and C are (N13, O6, O11, and O9), (O12, O1, N17, and O22), and (O1, O8, N25, and O20), respectively. All hydrogen atoms in A, B, and C are positively charged, according to the findings. The fact that (N13), (O10 and N17), and (O8 and N25) atoms are electron-withdrawing means that the (H38), (H26 and H45), and (H34 and H51) hydrogen atoms in compounds A, B, and C have the largest positive charges in contrast to other hydrogen atoms.

### 4.4 Natural bond orbital analysis

The NBO technique is regarded as a quick method for comprehending the characteristics of the electronic structure. As a straightforward framework for analyzing charge transfer, delocalization, and conjugative interactions in molecules, it is also a useful technique for assessing interactions between donors and acceptors (Weinhold and Landis, 2001).

The NBO analysis tool is a useful method for investigating both intra- and intermolecular bonding. It can effectively provide insights into charge transfer and hyper-conjugative interactions. In particular, NBO 5.0 software is utilized to compute electron density, rehybridization, and intramolecular charge delocalization within molecules. Furthermore, the NBO approach allows for quantitative analysis of bonding and anti-bonding interactions caused by second-order perturbation, expressed as perturbation energies E(2) by Refs. E^(2)^ = ΔE_ij_ = qi (F (i,j)^2^/E_j_−E_i_), where Ei and Ej are the diagonal elements, qi is donor orbital occupancy, and Fi,j is the NBO off-diagonal matrix element.

For both compounds, the computed and listed interactions between the Lewis-type occupied NBO orbital (bonding) and non-Lewis unoccupied NBO orbital (anti-bonding) are shown in Supplementary Table 11. There are only two types of donors, namely σ and π, and two types of acceptors, namely σ and π, according to the local inspection of the various donors and acceptors. According to observations of perturbation energy E^(2)^ for various transitions between these donors and acceptors, the following transitions for A molecule are extremely likely to occur: C16-C17→C14-C15 (96.87 kj/mol, π→π*), C16-C17→C18-C19 (63.97 kj/mol, π→π*), O9→C8-O11 (29.54 kj/mol, LP→π*), O11→C8-O9 (29.44 kj/mol, LP→π*), and N13→C14-C15 (63.97 kj/mol, LP→π*); for B molecule: C20-C21→C18-C19 (119.04 kj/mol, π→π*), C20-C21→C24-C25 (84.09 kj/mol, π→π*), N17→C18-C19 (33.29 kj/mol, LP→π*), N17→C15-C16 (29.78 kj/mol, LP→π*), and O22→C20-C21 (25.43 kj/mol, LP→π*); and for C molecule: C19-C22→C23-C24 (122.59 kj/mol, π→π*), C19-C22→C17-C18 (84.86 kj/mol, π→π*), N25→C23-C24 (31.97 kj/mol, LP→π*), N25→C13-C14 (28.54 kj/mol, LP→π*), O20→C19-C22 (25.49 kj/mol, LP→π*). These are the most probable transitions.

These transitions show stronger electron density, with strong intramolecular hyperconjugative interactions contributing more. Strong intramolecular interactions between the lone pairs (O9 to π* C8-O11, O11 to π* C8-O9, and N13 to π* C14-C15), (N17 to π* C18-C19, N17 to π* C15-C16, and O22 to π* C20-C21), and (N25 to π* C23-C24 and O20 to π* C19-C22) were also revealed by the NBO analysis. These interactions result in intramolecular charge transfer (ICT), which stabilizes the system.

## 5 Conclusion

The DME study of the three tested compounds revealed their ability to penetrate the BBB, and all showed a high potential for absorption from the GIT. Gardflorine A was the compound identified as a non-substrate for P-glycoprotein. All are lead-like molecules with no violation of the Lipinski rule of 5. In addition, the multi-target prediction showed that delavirdine could target 29 HCV-affecting proteins and 14 HIV-affecting proteins, while gardflorine A could target 53 proteins affecting HIV and 21 proteins affecting HCV. Gardflorine B targeted five HCV-affecting proteins and three HIV HIV-affecting proteins, while gardflorine C targeted nine HCV-affecting proteins and four HIV-affecting proteins. The docking study of gardflorine A, gardflorine B, and gardflorine C, with docking scores of −7.35, −7.64, and −7.56 kcal/mol, respectively, showed that they are also docked to the same binding site of HCV RNA-polymerase enzyme as delavirdine. The three natural indole compounds showed pi–alkyl interactions with the crucial amino acid Cys366. All compounds demonstrated interactions at the conserved palm site, with delavirdine displaying the strongest binding affinity. However, the natural indole compounds, particularly Gardflorine B, showed promising binding properties and inhibition potential, making them valuable candidates for further optimization and therapeutic development. The docking study results of HCV NS3/4A protease revealed the potential of these compounds as effective NS3/4A protease inhibitors. Among the compounds, gardflorine A demonstrated the strongest binding affinity and inhibitory potential, making it a promising candidate for targeting HIV-1 protease, HIV-1 RT, and HIV-1 integrase. The FMO analysis and chemical reactivity showed that the molecular structures of the compounds fall into the category of “high electrophiles,” with low values of ΔE_gap_, indicating high chemical reactivity. The calculated atomic charges using NBO analysis revealed both positive and negative charges in carbon atoms. Furthermore, the NBO analysis also showed strong intramolecular interactions between the lone pairs resulting in intramolecular charge transfer (ICT), which stabilizes the system. This study’s results provide new insights for developing drugs targeting HCV and HIV using molecular docking techniques and chemical reactivity analysis. Gardflorine B and C showed more selectivity toward HCV proteins than HIV targets compared to gardflorine A.

## Data Availability

All data are provided in the article and the supplementary files.
