# Supplementary material for: Exploring the potential of some natural indoles as antiviral agents: quantum chemical analysis, inverse molecular docking, and affinity calculations
Source: Front Chem. 2025 Jan 16;12:1521298. doi: 10.3389/fchem.2024.1521298 (PMC11779707; doi:10.3389/fchem.2024.1521298)
Supplement: Supplementary file 2 [file DataSheet1.pdf]

The Potential of some Natural Indoles against HIV and HCV: Inverse molecular docking, ADMET and quantum chemical studies

Appendix

Tab.S1	Protein targets in HIV and HCV, PdbID, ligand names, ligand similarity and docking scores of Delavirdine.
Tab.S2	Protein targets in HIV and HCV, Pdb ID, ligand names, ligand similarity and docking scores of the monoterpenoid indole gardflorine A
Tab.S3	Protein targets in HIV and HCV, PdbID, ligand names, ligand similarity and docking scores of the monoterpenoid indole gardflorine B.
Tab.S4	Protein targets in HIV and HCV, PdbID, ligand names, ligand similarity and docking scores of the monoterpenoid indole gardflorine C.
Tab.S5	Binding mode of delavirdine, gardflorine A, gardflorine B and gardflorine C in the active site of HCV-Polymerase enzyme
Tab.S6	Binding mode of delavirdine, gardflorine A, gardflorine B and gardflorine C in the active site of HCV-Protease enzyme.
Tab.S7	Binding mode of delavirdine, gardflorine A, gardflorine B and gardflorine C in the active site of HIV-Protease enzyme.
Tab.S8	Binding mode of delavirdine, and gardflorine A, in the active site of HIV-reverse transcriptase enzyme.
Tab.S9	Binding mode of gardflorine A, gardflorine B and gardflorine C in the active site of HIV-Integrase enzyme.

Table S1 : Protein targets in HIV and HCV, PdbID, ligand names, ligand similarity and docking scores of Delavirdine.

PDB	Target Class	Target Name	Ligand Name	Ligand Similarity Score	PSOVina2 Docking Score (kcal/mol) ▼
3vqs	HCV	RNA polymerase	JT1	0.442	-7.83
3h98	HCV	RNA polymerase	B5P	0.488	-7.713
3h5s	HCV	RNA polymerase	H5S	0.436	-7.593
3u4r	HCV	RNA polymerase	08F	0.489	-7.519

3cwj	HCV	RNA polymerase	321	0.478	-7.453
3gyn	HCV	RNA polymerase	B42	0.407	-7.364
3cde	HCV	RNA polymerase	N3H	0.426	-7.301
3upi	HCV	RNA polymerase	0C2	0.424	-7.169
4mib	HCV	RNA polymerase	28M	0.407	-7.165
3igv	HCV	RNA polymerase	B80	0.434	-7.13
4mk8	HCV	RNA polymerase	28Q	0.415	-7.083
3hky	HCV	RNA polymerase	IX6	0.407	-7.075
3uph	HCV	RNA polymerase	0C1	0.422	-7.01
3gnw	HCV	RNA polymerase	XNC	0.408	-7.002
3co9	HCV	RNA polymerase	3MS	0.43	-6.923
3d28	HCV	RNA polymerase	B34	0.405	-6.897
3h5u	HCV	RNA polymerase	H5U	0.418	-6.811
3fqk	HCV	RNA polymerase	79Z	0.426	-6.713
3cvk	HCV	RNA polymerase	N34	0.404	-6.683
3tyv	HCV	RNA polymerase	HI3	0.415	-6.626
3fql	HCV	RNA polymerase	79Z	0.426	-6.581
3g86	HCV	RNA polymerase	T18	0.432	-6.258
3hkw	HCV	RNA polymerase	IX6	0.407	-7.225
3hhk	HCV	RNA polymerase	77Z	0.433	-7.644
2fvc	HCV	RNA polymerase	888	0.437	-6.648
4nwk	HCV	NS3 protease	2R8	0.433	-5.981
3su6	HCV	NS3 protease	SU3	0.411	-5.879
5etx	HCV	NS3 protease	5RS	0.423	-6.691

5eqq	HCV	NS3 protease	5RS	0.423	-6.307
3gi4	HIV	Protease	K60	0.408	-6.08
4zip	HIV	Protease	G64	0.406	-7.804
3gi6	HIV	Protease	D78	0.401	-5.86
2qi5	HIV	Protease	MZ7	0.409	-6.753
2pk5	HIV	Protease	075	0.411	-7.084
2i0d	HIV	Protease	MUT	0.408	-6.039
3gi5	HIV	Protease	K62	0.42	-6.008
3mxd	HIV	Protease	K53	0.414	-6.297
3mxe	HIV	Protease	K54	0.417	-5.848
1zp8	HIV	Protease	AB2	0.416	-5.981
3i0r	HIV	Reverse transcriptase	RT3	0.412	-8.157
2rf2	HIV	Reverse transcriptase	MRX	0.461	-7.949
3i0s	HIV	Reverse transcriptase	RT7	0.403	-7.408
4i7f	HIV	Reverse transcriptase	NVE	0.403	-7.913

Table S2 : Protein targets in HIV and HCV, Pdb ID, ligand names, ligand similarity and docking scores of the monoterpenoid indole gardflorine A

	PDB	Target Class	Target Name	Ligand Name	Ligand Similarity Score	PSOVina2 Docking Score (kcal/mol) ▾
1	3upi	HCV	RNA polymerase	0C2	0.405	-7.357
2	3h5s	HCV	RNA polymerase	H5S	0.434	-6.81
3	3cwj	HCV	RNA polymerase	321	0.407	-6.708

4	3gnw	HCV	RNA polymerase	XNC	0.437	-6.848
5	3q0z	HCV	RNA polymerase	23E	0.432	-6.118
6	2yoj	HCV	RNA polymerase	8Y6	0.435	-6.99
7	3cso	HCV	RNA polymerase	XNI	0.443	-6.975
8	3hky	HCV	RNA polymerase	IX6	0.447	-6.645
9	3gol	HCV	RNA polymerase	XND	0.423	-6.576
10	4nld	HCV	RNA polymerase	2N7	0.412	-6.398
11	3hkw	HCV	RNA polymerase	IX6	0.447	-7.12
12	3ske	HCV	RNA polymerase	054	0.41	-6.977
13	3hhk	HCV	RNA polymerase	77Z	0.407	-6.975
21	2dxs	HCV	RNA polymerase	JTP	0.428	-6.482
14	5etx	HCV	NS3 protease	5RS	0.426	-6.231
15	2xcn	HCV	NS3 protease	C8D	0.411	-5.832
16	2xni	HCV	NS3 protease	TR8	0.424	-5.59
17	3p8n	HCV	NS3 protease	L4T	0.451	-5.033
18	3p8o	HCV	NS3 protease	L5T	0.449	-4.897
19	4nwk	HCV	NS3 protease	2R8	0.401	-5.809
20	3su6	HCV	NS3 protease	SU3	0.436	-6.66
1	3ok9	HIV	Protease	G52	0.407	-9.722
2	2qd8	HIV	Protease	065	0.401	-8.203
3	2pk5	HIV	Protease	075	0.426	-8.124
4	3a2o	HIV	Protease	KNJ	0.401	-8.921
5	2qci	HIV	Protease	065	0.401	-7.933
6	2z4o	HIV	Protease	065	0.401	-7.861

7	2qd6	HIV	Protease	065	0.401	-7.683
8	3kdd	HIV	Protease	JZQ	0.438	-7.366
9	3kdc	HIV	Protease	JZP	0.437	-7.331
10	4kb9	HIV	Protease	G79	0.401	-7.301
11	2qd7	HIV	Protease	065	0.401	-7.153
12	2wl0	HIV	Protease	5AH	0.417	-6.961
13	3vfa	HIV	protease	031	0.41	-6.953
14	2pk6	HIV	Protease	O33	0.443	-6.941
15	3vfb	HIV	Protease	031	0.41	-6.931
16	2wkz	HIV	Protease	5AH	0.417	-6.894
17	3kdb	HIV	Protease	006	0.445	-6.813
18	2q54	HIV	Protease	MU1	0.404	-6.78
19	3vf5	HIV	Protease	031	0.41	-6.707
20	3o9h	HIV	Protease	K2E	0.404	-6.506
21	3vf7	HIV	protease	031	0.41	-6.403
22	4fe6	HIV	Protease	0TQ	0.444	-6.39
23	2q55	HIV	Protease	MU0	0.426	-6.248
24	3gi5	HIV	Protease	K62	0.403	-5.981
25	3o9i	HIV	Protease	A61	0.412	-5.803
26	4he9	HIV	Protease	G52	0.407	-9.313
27	4hdf	HIV	Protease	G52	0.407	-8.109
28	4heg	HIV	Protease	G52	0.407	-8.082
29	4hdb	HIV	Protease	G52	0.407	-8.464
30	4hdp	HIV	Protease	G52	0.407	-8.463

31	2uy0	HIV	Protease	HV1	0.409	-6.733
32	1hpx	HIV	Protease	KNI	0.407	-6.715
33	3h5b	HIV	Protease	031	0.41	-6.584
34	3mxd	HIV	Protease	K53	0.405	-6.037
35	3mxe	HIV	Protease	K54	0.412	-6.117
36	3ggv	HIV	Protease	GGV	0.43	-6.253
37	4u7q	HIV	Protease	3EM	0.401	-5.866
38	5dgu	HIV	Protease	5B7	0.401	-7.459
39	2cen	HIV	Protease	4AH	0.409	-6.602
40	2cem	HIV	Protease	2AH	0.411	-6.344
41	1mrx	HIV	Protease	K57	0.417	-6.897
42	1mrw	HIV	Protease	K57	0.417	-6.332
43	3o9d	HIV	Protease	K19	0.415	-5.824
44	3o9c	HIV	Protease	K20	0.407	-5.565
45	3o9a	HIV	Protease	K14	0.401	-5.754
46	3qo9	HIV	Reverse transcriptase	QO9	0.409	-8.389
47	4i7f	HIV	Reverse transcriptase	NVE	0.427	-8.219
48	3nf6	HIV	Integrase	IMV	0.401	-6.079
59	4lh5	HIV	Integrase	LF0	0.403	-4.669
50	5kgw	HIV	Integrase	7SK	0.449	-4.263
51	5kgx	HIV	Integrase	7SK	0.449	-4.107
52	3nf8	HIV	Integrase	CDQ	0.403	-6.04
53	4ojr	HIV	Integrase	2SQ	0.403	-4.508

Table S3 : Protein targets in HIV and HCV, PdbID, ligand names, ligand similarity and docking scores of the monoterpenoid indole gardflorine B.

PDB	Target Class	Target Name	Ligand Name	Ligand Similarity Score	PSOVina2 Docking Score (kcal/mol)
3hkw	HCV	RNA polymerase	IX6	0.408	-7.647
3hky	HCV	RNA polymerase	IX6	0.408	-7.357
3p8o	HCV	NS3 protease	L5T	0.424	-5.605
4nwk	HCV	NS3 protease	2R8	0.401	-6.156
3p8n	HCV	NS3 protease	L4T	0.417	-5.16
5kgw	HIV	Integrase	7SK	0.404	-4.827
5kgx	HIV	Integrase	7SK	0.404	-4.151
2pk6	HIV	Protease	O33	0.401	-5.899

Table S4 : Protein targets in HIV and HCV, PdbID, ligand names, ligand similarity and docking scores of the monoterpenoid indole gardflorine C.

PDB	Target Class	Target Name	Ligand Name	Ligand Similarity Score	PSOVina2 Docking Score (kcal/mol)
5eqq	HCV	NS3 protease	5RS	0.402	-7.03
4nwk	HCV	NS3 Protease	2R8	0.408	-5.206
3p8n	HCV	NS3 Protease	L4T	0.425	-5.412
3p8o	HCV	NS3 Protease	L5T	0.432	-5.113
2xcn	HCV	NS3 protease	C8D	0.411	-4.952

5etx	HCV	NS3 protease	5RS	0.402	-6.273
3hkw	HCV	RNA polymerase	IX6	0.419	-7.56
3hky	HCV	RNA polymerase	IX6	0.419	-7.551
3gnw	HCV	RNA polymerase	XNC	0.404	-7.046
2pk5	HIV	Protease	075	0.405	-7.447
2pk6	HIV	Protease	O33	0.412	-6.593
5kgx	HIV	Integrase	7SK	0.404	-4.441
5kgw	HIV	Integrase	7SK	0.404	-4.598

Table S5. Binding mode of delavirdine, gardflorine A, gardflorine B and gardflorine C in the active site of HCV-Polymerase enzyme

**HCV Polymerase**				
	**Distance**	**Type of interaction**	**Amino acids**	**Docking Score**
Delavirdine<br><br>PDB ID:<br>3vqs	2.81468<br>3.29776<br>3.42476<br>4.86108<br>5.25674<br>5.39171	Conventional Hydrogen Bond<br>Carbon Hydrogen Bond<br>Carbon Hydrogen Bond<br>Pi-Pi T-shaped<br>Pi-Pi T-shaped<br>Pi-Alkyl	A:LEU547:O<br>A:PHE193:O<br>A:TYR452:OH<br>A:TYR448<br>A:PHE193<br>A:CYS366	-7.83
Gardflorine A<br><br>PDB ID: 3upi	3.38861<br>3.91496<br>4.19742<br>4.91361<br>4.97536	Conventional Hydrogen Bond<br>Alkyl<br>Alkyl<br>Pi-Alkyl<br>Pi-Alkyl	A:ASN316:ND2<br>A:MET414<br>A:CYS366<br>A:PHE193<br>A:TYR415	-7.357
Gardflorine B<br>PDB ID: 3hkw	2.97212<br>4.14719<br>2.6594<br>2.96741<br>5.64017<br>5.11649<br>4.15778<br>5.16223<br>4.57651	Salt Bridge<br>Attractive Charge<br>Conventional Hydrogen Bond<br>Carbon Hydrogen Bond<br>Pi-Pi T-shaped<br>Pi-Pi T-shaped<br>Alkyl<br>Pi-Alkyl<br>Pi-Alkyl	A:ARG386:NH1<br>A:ARG394:NH1<br>A:CYS366:O<br>A:ARG200:CD<br>A:PHE415<br>A:PHE415<br>A:CYS366<br>A:CYS366<br>A:CYS366	-7.647

Gardflorine C<br><br>PDB ID: 3hkw	3.07877<br>2.769<br>5.41762<br>4.86605<br>4.51732<br>5.01792<br>5.06717	Conventional Hydrogen Bond<br>Conventional Hydrogen Bond<br>Pi-Alkyl<br>Pi-Alkyl<br>Pi-Alkyl<br>Pi-Alkyl<br>Pi-Pi T-shaped	A:ASN411:N<br>A:SER407:O<br>A:CYS366<br>A:MET414<br>A:CYS366<br>A:MET414<br>A:TYR448	-7.56

Table S6. Binding mode of delavirdine, gardflorine A, gardflorine B and gardflorine C in the active site of HCV-Protease enzyme.

HCV Protease				
	**Distance**	**Type of interaction**	**Amino acids**	**Docking scores**
Delavirdine<br>PDB ID: 5etx	2.92519<br>3.07665<br>3.69735<br>4.16189<br>5.15037<br>5.25271	Conventional Hydrogen Bond<br>Conventional Hydrogen Bond<br>Pi-Donor Hydrogen Bond<br>Pi-Alkyl<br>Pi-Alkyl<br>Pi-Alkyl	A:SER159:N<br>A:ALA157:N<br>A:ALA157:N<br>A:ALA157<br>A:HIS57<br>A:ALA157	-6.691
Gardflorine A<br>PDB ID: 5etx	3.20148<br>4.59948<br>4.26789<br>4.25017	Conventional Hydrogen Bond<br>Alkyl<br>Alkyl<br>Pi-Alkyl	A:HIS57:NE2<br>A:ALA156<br>A:ALA157<br>A:HIS57	-6.231
Gardflorine B<br>PDB ID: 4nwk	3.60142<br>3.39425<br>3.3786<br>2.79258<br>3.23479<br>5.23747<br>5.13105	Carbon Hydrogen Bond<br>Conventional Hydrogen Bond<br>Conventional Hydrogen Bond<br>Conventional Hydrogen Bond<br>Conventional Hydrogen Bond<br>Pi-Alkyl<br>Pi-Alkyl	A:LEU135:O<br>A:THR42:N<br>A:SER139:N<br>A:SER139:OG<br>A:THR42:O<br>A:LYS136<br>A:ALA157	-6.156
Gardflorine C<br>PDB ID: 2pk5	3.06998<br>3.70786<br>3.89257<br>3.86755<br>4.67404<br>3.92514<br>2.8018<br>3.25636<br>4.17866<br>4.68185<br>4.24978<br>4.72186<br>5.44642<br>4.67241	Carbon Hydrogen Bond<br>Carbon Hydrogen Bond<br>Pi-Cation;Pi-Donor Hydrogen Bond<br>Pi-Cation;Pi-Donor Hydrogen Bond<br>Pi-Pi T-shaped<br>Pi-Pi T-shaped<br>Alkyl<br>Alkyl<br>Alkyl<br>Alkyl<br>Pi-Alkyl<br>Pi-Alkyl<br>Pi-Alkyl<br>Pi-Alkyl	A:LYS136:CA<br>A:THR 42:O<br>A:LYS136:NZ<br>A:LYS136:NZ<br>A:HIS57<br>A:HIS57<br>A:ALA157<br>A:ILE132<br>A:LEU135<br>A:CYS159<br>A:LYS136<br>A:ALA139<br>A:LYS136<br>A:ALA139	-7.447

Table S7. Binding mode of delavirdine, gardflorine A, gardflorine B, and gardflorine C in the active site of HIV-Protease enzyme.

HIV Protease				
	Distance	Type of interaction	Amino acids	**Docking scores**
Delavirdine<br>PDB ID: **4zip**	3.34319<br>3.76766<br>3.86979<br>3.73655<br>5.15524<br>5.42037	Conventional Hydrogen Bond<br>Carbon Hydrogen Bond<br>Pi-Sigma<br>Pi-Sigma<br>Alkyl<br>Alkyl	B:GLY27:O<br>B:PRO81:CD:B<br>B:ALA28:CB<br>B:ILE47:CG2<br>B:ALA28<br>B:ILE84	-7.804
Gardflorine A<br>PDB ID: **3ok9**	3.17203<br>3.36557<br>2.9579<br>3.66437<br>4.49408<br>5.0104<br>5.11552<br>3.96855<br>5.37352<br>4.81322<br>4.39743<br>5.37422<br>5.18117	Carbon Hydrogen Bond<br>Carbon Hydrogen Bond<br>Carbon Hydrogen Bond<br>Carbon Hydrogen Bond<br>Alkyl<br>Alkyl<br>Alkyl<br>Alkyl<br>Alkyl<br>Alkyl<br>Alkyl<br>Alkyl<br>Pi-Alkyl	A:GLY49:CA<br>A:GLY49:CA:B<br>A:GLY48:O:B<br>A:ILE47:O<br>A:ALA28<br>A:PRO81<br>A:ILE54<br>A:ILE54<br>A:VAL32<br>A:ILE47<br>A:PRO81<br>A:VAL82<br>A:PRO81	-9.722
Gardflorine B<br>PDB ID: **2pk6**	4.18172<br>4.18396<br>2.83923<br>3.18433<br>3.60331<br>3.53184<br>3.8176<br>4.92188<br>5.48268<br>5.46123	Attractive Charge<br>Attractive Charge<br>Conventional Hydrogen Bond<br>Conventional Hydrogen Bond<br>Carbon Hydrogen Bond<br>Pi-Sigma<br>Pi-Sigma<br>Pi-Alkyl<br>Pi-Alkyl<br>Pi-Alkyl	B:ASP29:OD2<br>B:ASP30:OD2<br>B:ASP29:N<br>B:THR80:OG1<br>B:MET46:O<br>B:ILE47:CD1<br>B:ILE47:CD1<br>B:PHE53<br>B:VAL32<br>B:ILE54	-5.899
Gardflorine C<br>PDB ID: **2pk5**	5.43189<br>2.53277<br>2.87307<br>3.54425<br>4.70935<br>4.14062<br>4.5502<br>4.29476<br>5.22278	Attractive Charge<br>Conventional Hydrogen Bond<br>Conventional Hydrogen Bond<br>Carbon Hydrogen Bond<br>Pi-Anion<br>Pi-Anion<br>Alkyl<br>Pi-Alkyl<br>Pi-Alkyl	A:ASP30:OD2<br>A:GLY48:O<br>A:GLY48:O<br>A:ASP30:O<br>A:ASP29:OD2<br>A:ASP29:OD2<br>A:ILE84<br>A:ILE47<br>A:ILE47	-7.447

Table S8. Binding mode of delavirdine, and gardflorine A, in the active site of HIV-reverse transcriptase enzyme.

HIV-reverse transcriptase				
	Distance	Type of interaction	Amino acids	Docking scores

Delavirdine<br>PDB ID: 3i0r	3.39332<br>3.87863<br>3.64137<br>3.87804<br>3.50477<br>3.61314<br>3.95989<br>5.78939<br>4.48149<br>4.60806<br>4.47467<br>4.47028<br>4.49435<br>5.15994<br>4.3315<br>5.33352	Conventional Hydrogen Bond<br>Pi-Cation;Pi-Donor Hydrogen Bond<br>Pi-Cation;Pi-Donor Hydrogen Bond<br>Pi-Sigma<br>Pi-Sigma<br>Pi-Sigma<br>Pi-Sigma<br>Pi-Pi Stacked<br>Alkyl<br>Alkyl<br>Pi-Alkyl<br>Pi-Alkyl<br>Pi-Alkyl<br>Pi-Alkyl<br>Pi-Alkyl<br>Pi-Alkyl	A:LYS103:NZ<br>A:LYS101:NZ<br>A:LYS101:NZ<br>A:VAL179:CG2<br>A:TYR188<br>A:TRP229<br>A:TRP229<br>A:TYR188<br>A:LEU100<br>A:VAL179<br>A:LYS101<br>A:LYS101<br>A:VAL179<br>A:LEU100<br>A:VAL106<br>A:LEU234	-8.157
Gardflorine A<br>PDB ID: 3qo9	3.91597<br>3.58117<br>4.32884<br>4.10255<br>4.34449<br>5.30961<br>4.93029<br>4.70686<br>4.04282<br>5.14169	Pi-Donor Hydrogen Bond<br>Pi-Sigma<br>Pi-Pi Stacked<br>Alkyl<br>Alkyl<br>Alkyl<br>Pi-Alkyl<br>Pi-Alkyl<br>Pi-Alkyl<br>Pi-Alkyl	A:TYR318:OH<br>A:TRP229<br>A:TYR318<br>A:LEU100<br>A:VAL106<br>A:LEU234<br>A:TYR181<br>A:TYR188<br>A:TRP229<br>A:LYS102	-8.389

Table S9. Binding mode of gardflorine A, gardflorine B and gardflorine C in the active site of HIV-Integrase enzyme.

HIV-integrase				
	Distance	Type of interaction	Amino acids	Docking scores
Gardflorine A<br>PDB ID: 3nf6	3.21699<br>3.71775<br>3.46787<br>5.49934<br>4.95791<br>4.87349<br>5.46493	Conventional Hydrogen Bond<br>Carbon Hydrogen Bond<br>Carbon Hydrogen Bond<br>Alkyl<br>Pi-Alkyl<br>Pi-Alkyl<br>Pi-Alkyl	A:TYR83:OH<br>A:TYR83:OH<br>A:GLU85:OE2<br>A:VAL180<br>A:TYR83<br>A:PHE181<br>A:HIS185	-6.079
Gardflorine B<br>PDB ID: 5kgw	3.68203<br>3.50106<br>3.75076	Conventional Hydrogen Bond<br>Pi-Donor Hydrogen Bond<br>Pi-Sigma	A:MET178:SD<br>A:THR174:OG1<br>A:THR174:CG2	-4.827
Gardflorine C<br>PDB ID: 5kgw	3.28969<br>3.29414<br>3.12858<br>3.38254<br>3.14572<br>3.26189<br>3.97062<br>4.15965	Conventional Hydrogen Bond<br>Conventional Hydrogen Bond<br>Conventional Hydrogen Bond<br>Carbon Hydrogen Bond<br>Carbon Hydrogen Bond<br>Carbon Hydrogen Bond<br>Pi-Sulfur<br>Pi-Alkyl	A:GLU170:N<br>A:HIS171:N<br>A:THR174:OG1<br>A:HIS171:CD2<br>A:GLN168:O<br>A:GLN168:O<br>A:MET178:SD<br>A:MET178	-4.598

Docking Validation

The docking methodology was rigorously validated through a re-docking and superimposition process involving the native ligands which was originally co-crystallized with the active site of the target proteins (pdb ID: 2PK5, 2PK6, 3HKW, 3I0R, 3NF6, 3OK9, 3QO9, 3UPI, 3VQS, 4NWK, 4ZIP, 5EQQ, 5ETX, and 5KGW). During the validation, the binding pose of this co-crystallized ligand was accurately reproduced, ensuring that the docking protocol was reliable. The superimposition of the re-docked ligand with the native ligand demonstrated a high degree of alignment, confirming that the docking procedure could precisely mimic the original ligand's interactions within the active site. This successful validation, depicted in Fig. S.X, underscores the robustness and accuracy of the docking protocol, making it suitable for predicting the binding modes of other compounds in subsequent studies.

2pk5	2pk6	3hkw
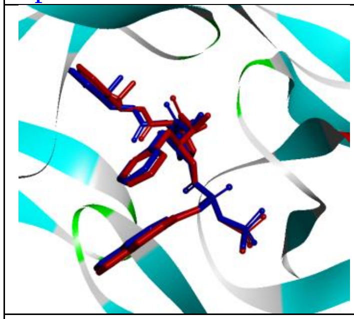	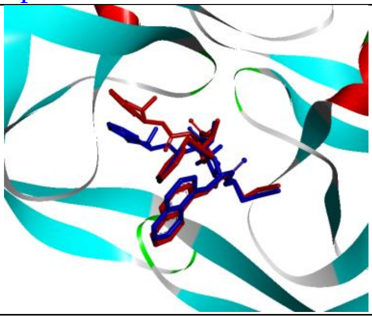	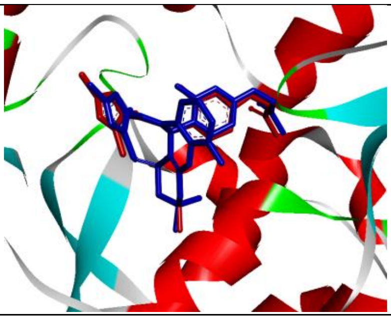
3i0r	3nf6	3ok9
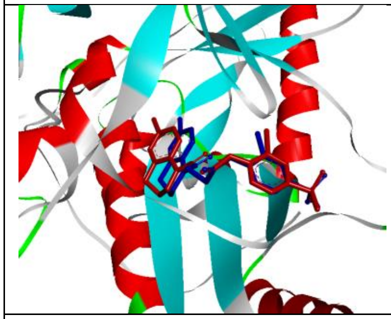	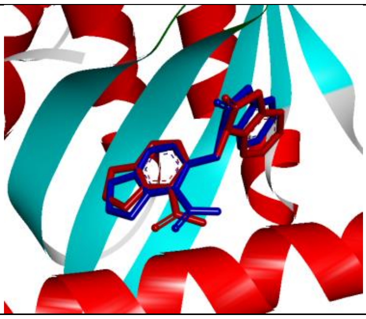	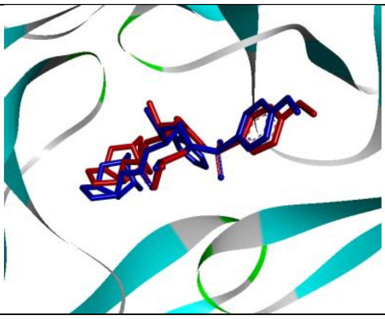
3qo9	3upi	3vqs

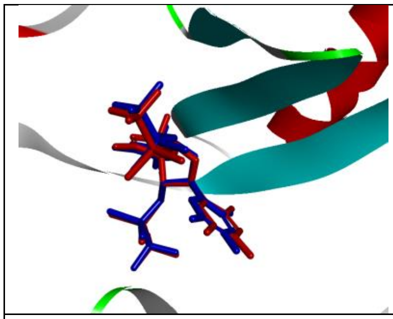	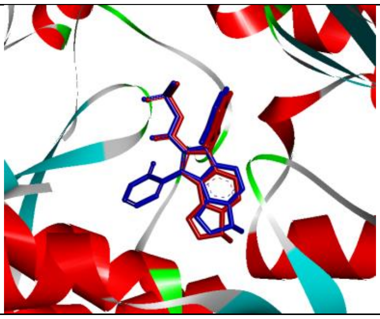	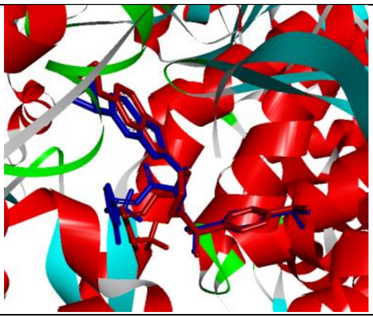
4nwk	4zip	5eqq
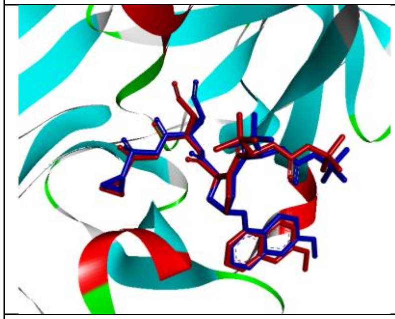	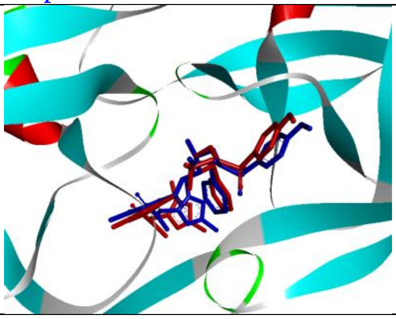	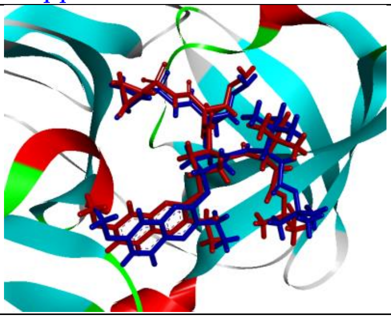
5etx	5kgw	
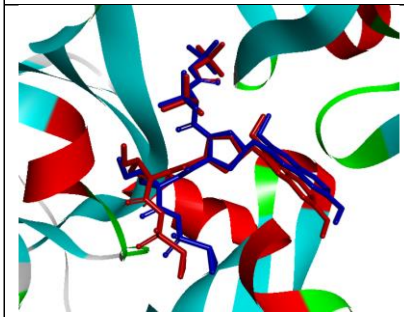	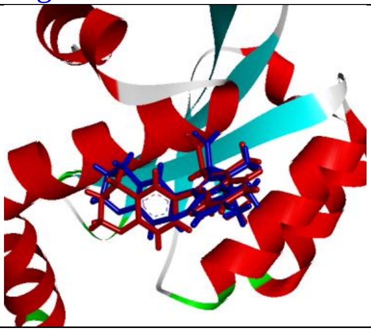	

Fig. (S.X): 3D representation of the superimposition of the co-crystallized (blue) and the redocking pose (red) of the cocrystallized ligands in target proteins (pdb ID: 2PK5, 2PK6, 3HKW, 3I0R, 3NF6, 3OK9, 3QO9, 3UPI, 3VQS, 4NWK, 4ZIP, 5EQQ, 5ETX, and 5KGW).
